# Mismatches between the genetic and phenotypic sex in the wild Kou population of Nile tilapia *Oreochromis niloticus*

**DOI:** 10.7717/peerj.7709

**Published:** 2019-09-18

**Authors:** Rokyatou Sissao, Helena D’Cotta, Jean-François Baroiller, Aboubacar Toguyeni

**Affiliations:** 1 Unité de recherche aquaculture et biodiversité aquatique/Laboratoire d’études et de recherche sur les ressources naturelles et sciences de l’environnement, Université Nazi BONI, Bobo-Dioulasso, Burkina Faso; 2 Institut de l’environnement et de recherches agricoles, Centre national de la recherche scientifique et technologique, Bobo-Dioulasso, Burkina Faso; 3 Centre international de recherche-développement sur l’élevage en zone subhumide, Bobo-Dioulasso, Burkina Faso; 4 ISEM, Université de Montpellier, CNRS, IRD, EPHE, Montpellier, France; 5 UMR ISEM, CIRAD, Montpellier, France

**Keywords:** Nile tilapia, Sex determination, Sex-reversal, Sex chromosomes, Wild population

## Abstract

Sex determination and sex chromosomes can be very diverse between teleost species. The group of tilapias shows a polymorphism in sex determination not only between closely related species but also between domestic strains within a species. In the Nile tilapia, the major effect genes and therefore the Y chromosome have been located on either linkage group 1 (LG1) or LG23 depending on the strains. In a Japanese strain, the sex determinant of LG23 (the *amhY* gene) has been identified as a duplicated *amh* (anti-Müllerian hormone) gene, with its gametolog found on the X chromosome (*amhX*). *AmhY* is located in tandem with the *amhΔY* gene (a truncated form) on the Y chromosome. X and Y chromosome markers based on the *amh* genes have been validated only on a few domestic strains but not in wild populations. Here, we used four of these markers in order to examine (1) the possible variation in sex determination of a wild population of Nile tilapia living in Lake Kou (Burkina Faso), (2) putative polymorphisms for these *amh* copies and (3) the existence of sex reversed individuals in the wild. Our genotyping of 91 wild Kou individuals with the *amh* sex-diagnostic markers of LG23 showed that while phenotypic females were all XX, phenotypic males were either XY or XX. Progeny testing of eight of these XX males revealed that one of these males consistently sired all-female progenies, suggesting that it is a wild sex reversed male (which could result from high temperature effects). The other XX males gave balanced sex ratios, suggesting that sex is controlled by another locus (possibly on another LG) which may be epistatically dominant over the LG23 locus. Finally, identification of unexpected *amh* genotypes was found for two individuals. They produced either balanced or female-biased sex ratios, depending on the breeder with whom they were crossed, suggesting possible recombination between the X and the Y chromosomes.

## Introduction

Sex determination and sex differentiation in fish are of major interest for both basic and applied research since some species require sex control in order to inhibit reproduction, improve growth and/or flesh quality (Baroiller & D’Cotta, 2016; Baroiller & D’Cotta, 2018). In gonochoristic fish, an individual retains a unique sex throughout its life. In teleosts this sex can be determined by genetic factors (genetic sex determination = GSD) with sex chromosomes (XX/XY or ZZ/ZW) or without (polyfactorial systems), or by environmental factors (environment sex determination = ESD, usually temperature sex determination = TSD) (Devlin & Nagahama, 2002; Baroiller et al., 2009). However, as also demonstrated in reptiles (Sarre, Georges & Quinn, 2004), these are not exclusive systems but a *continuum* where both factors may interact in numerous fish species (Baroiller & D’Cotta, 2001; Devlin & Nagahama, 2002; Baroiller, D’Cotta & Saillant, 2009; Baroiller et al., 2009; Heule, Salzburger & Böhne, 2014). Contrary to mammals (Graves, 2006), fish sex chromosomes are mainly homomorphic and cannot be distinguished by karyotype analysis. In the medaka *Oryzias latipes* the male-determining gene *Dmy* (DM-domain gene on the Y chromosome) was found in a region of the Y chromosome showing only 258 kb suppressed recombination (Matsuda et al., 2002). Some fish male-determiners have only been distinguished by a few base pair (bp) substitutions compared to the X chromosome. *Oryzias luzonensis* males have the Y gonadal soma derived growth factor (*gsdf)* gene that differs in 12 bp synonymous substitutions, ensuring the high *gsdf* expression (Myosho et al., 2012). In the *Takifugu* genus males are heterozygous for a Y-specific allele located in the anti-Müllerian receptor 2 (*amhr2*) gene (Kamiya et al., 2012). In several fish species such as guppy *Poecilia reticulata*, medaka *O. latipes*, pejerrey *Odontesthes bonariensis*, goldfish *Carassius auratus*, rainbow trout *Oncorhynchus mykiss*, and Nile tilapia *Oreochromis niloticus*, gene content is so similar between the X and Y chromosomes that it allows the YY male to be viable and fertile (Devlin & Nagahama, 2002). Furthermore, the process of sex determination and sex differentiation in fish is extremely flexible leading to discordance between the sexual phenotype and the sexual genotype (Baroiller & D’Cotta, 2001; Devlin & Nagahama, 2002; Baroiller et al., 2009; Heule, Salzburger & Böhne, 2014; Baroiller & D’Cotta, 2016; Hattori et al., 2018).

The Nile tilapia *O. niloticus* (Linnaeus, 1758) is a major world aquaculture species where sex determination has been abundantly studied for sex control because males grow faster than females and mixed-sex farming leads to unwanted reproduction when fish become sexually mature (Beardmore, Mair & Lewis, 2001; Baroiller & D’Cotta, 2018). Previous studies revealed a complex sex determining system governed by interactions between genetic factors and temperature. Besides the effects of major genetic factors located on the sex chromosomes, minor genetic factors contributed by the two parents also contribute to the sex ratio (Baroiller & D’Cotta, 2001; Lee, Penman & Kocher, 2003; Baroiller, D’Cotta & Saillant, 2009; Baroiller et al., 2009; Baroiller & D’Cotta, 2016). It has a male heterogametic system (XX/XY), with an homomorphic sex chromosome pair (Majumdar & McAndrew, 1986; Cnaani et al., 2008; Poletto et al., 2010). Studies have revealed several sex-linked loci in Nile tilapia with the sex determining locus mapped to either LG1 (Lee, Penman & Kocher, 2003; Cnaani et al., 2008; Lee et al., 2011; Palaiokostas et al., 2013; Gammerdinger et al., 2014) or LG23 (Shirak et al., 2006; Eshel et al., 2011; Eshel et al., 2012; Sun et al., 2014; Li et al., 2015; Wessels et al., 2017) depending on the strains or families. A duplicated *amh* gene (*amhY*) is supposed to be the sex determining gene in Nile tilapia strains relying on LG23, the Y chromosome in these strains (Li et al., 2015).

The Anti-Müllerian hormone (*amh*) induces the regression of the Müllerian duct in male mammals and is expressed in Sertoli cells during testis differentiation (Josso, Di Clemente & Gouédard, 2001). Although this duct is absent in teleosts, they have orthologous *amh* genes which are also involved in gonad differentiation (Pfennig, Standke & Gutzeit, 2015). In the Nile tilapia, the sex determining *amhY* gene found in strains relying on the LG23 system is expressed ∼9 days post fertilization (dpf) in differentiating XY testes (Li et al., 2015). Previous studies (before the identification of the 3 *amh* copies) showed *amh* to be more strongly expressed in differentiating testes but at later stages ∼17 dpf (Ijiri et al., 2008; Poonlaphdecha et al., 2011) with an earlier dimorphic expression also shown in male brains at 10 to 15 dpf (Poonlaphdecha et al., 2011). *Amh* gene expression was rapidly up-regulated by masculinizing temperature (Poonlaphdecha et al., 2013) and found to belong to a major QTL involved in both autosomal and temperature-induced sex reversal (Wessels et al., 2014; Wessels et al., 2017).

The *amhY* gene on the Y chromosome in LG23 systems has been found to be located in tandem with another *amh* gene or copy now named *amh*Δ*Y* which lacks the TGF-β domain, identified first in the Swansea strain (Eshel et al., 2014) and later in a Japanese strain (Li et al., 2015). *AmhY* and *amh* Δ*Y* have also been shown to be associated to sex in a Manzala strain from the University of Göttingen (Wessels et al., 2017). Its X-chromosome homolog or gametolog (Li et al., 2015) is named *amhX* in our study. The *amhY* sequence possesses a missense SNP in exon 2 considered fundamental in the Japanese strain for testis determination (Li et al., 2015). Otherwise *amhX, amhY* and *amh* Δ*Y* genes are distinguishable by many insertions and deletions which were used as genotypic sex-chromosome markers in the Japanese strain.

Our group has been studying the *amh* sequences in other domestic strains but also in several wild populations of Nile tilapia from both Western and Eastern Africa. We have found by Sanger sequencing 12 SNPs in the *amh* genes that are strongly associated to the phenotypic sex (Table S1) but the diagnostic missense SNP in exon 2 (Li et al., 2015) was not polymorphic. These high polymorphisms within the *amh* sequences further reflects Nile tilapia variability in sex associations. Hence, it is very important to analyze the sex determining system not only in each domestic strain but also in wild populations to better understand its genetic basis.

The objectives of the current study was to use the available *amh* markers for the X and the Y chromosomes on wild-caught individuals of Nile tilapia from Lake Kou in Burkina Faso (West Africa), in order to examine (1) the possible variation in sex determination in this wild population, (2) putative polymorphisms for these *amh* genes and (3) the existence of sex reversed individuals in the wild.

## Materials & Methods

### Animals and sampling

**Figure 1:**
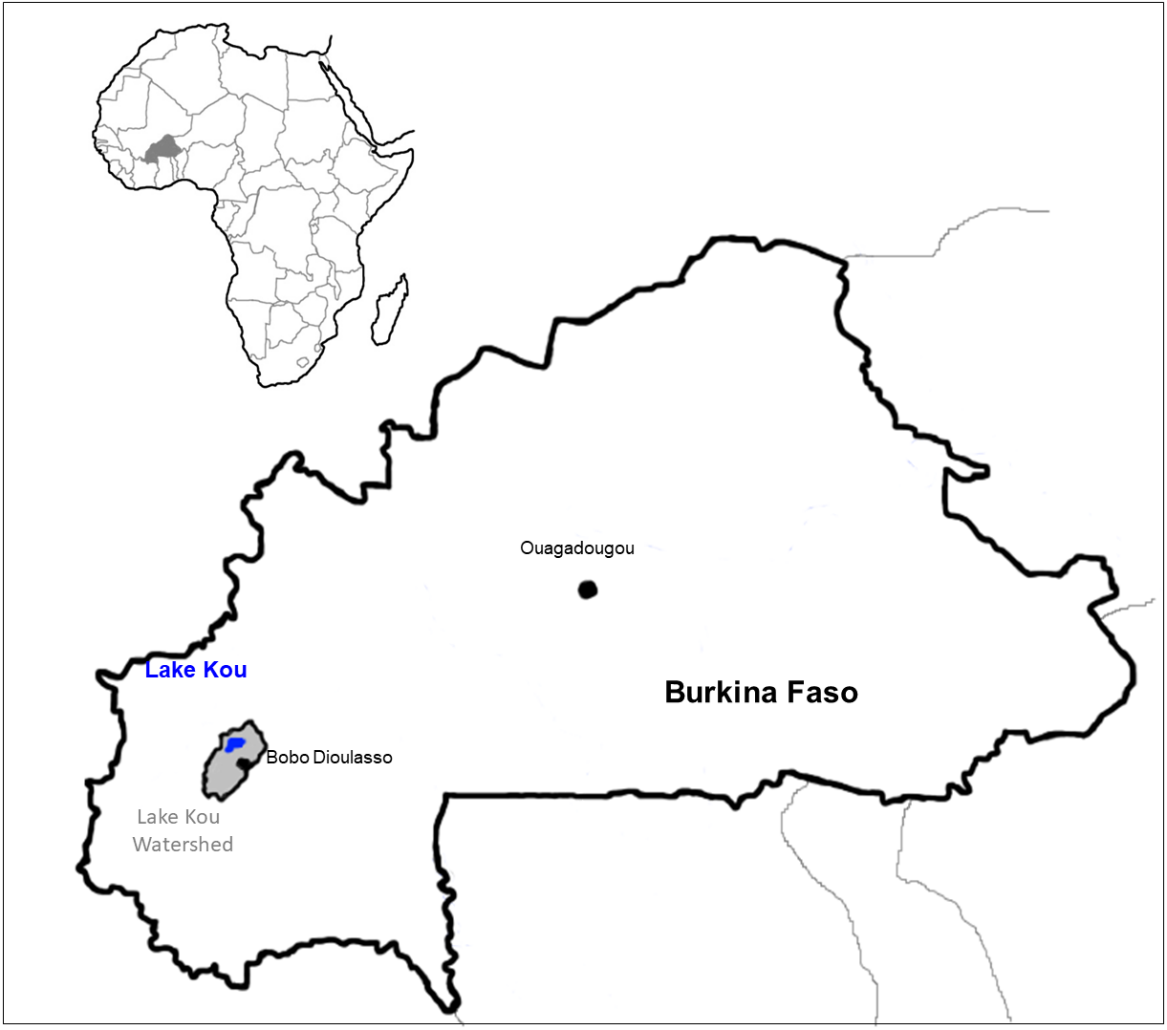
Lake Kou location in Burkina Faso. Burkina Faso is located in West Africa, with an enlargement showing Lake Kou location and its watershed.

Ninety one individuals (46 males and 45 females) were collected in Lake Kou (Bama, Burkina Faso) during July 2015 (Fig. 1). During the collecting period the water temperature ranged between 25.1 °C and 38.4 ± 2.1 °C and pH values were acidic varying between 5 and 6.7 ± 0.2 (Tables S2 and S3). The wild caught fish (G0 individuals) were transferred to the experimental facilities of the Aquaculture and Aquatic Biodiversity Research Unit (Nasso, Burkina Faso), stocked in concrete basins (open circuit) under natural photoperiod conditions and fed *ad libitum* with commercial pellets (Skretting, Norway). They were individually tagged with Passive Integrated Transponders (PIT tags EasyTracID) and the phenotypic sex was determined by the genital papilla (Baroiller & D’Cotta, 2018). A fragment of the caudal fin was sampled from each fish and stored in absolute ethanol (99%) until biomolecular analysis. To validate the sex-chromosome markers we also used in this study fin clips from males and females from the Japanese strain and known genotypes corresponding to XX females, XY males and YY males obtained from previous crossings from the Manzala-Tihange (Belgium) strain kept at Cirad. YY males have been obtained by the classic approach (see the review of Baroiller & D’Cotta, 2018). Briefly, the different steps are the following: hormonal feminization of classic progenies, identification of XY females by progeny testing, crossing an XY male with an XY female and finally identification of the YY male by progeny testing.

### Ethics statement

All animal procedures were performed in accordance to the French protocol No 2016101810463. Capture of wild Kou fish was conducted in accordance with articles 177, 187 and 202 from the Ministry of Animal Resources and Fisheries of Burkina Faso. Experimental procedures were under the Laboratory agreement for animal experimentation N° A-34-172-24, with the authorization to experiment N°35-15.

### Progeny testings

For the reproductions we stocked one tagged male with three tagged females per 300 L aquarium in recirculated and thermo-regulated systems. Each spawned female and fertilizing male were identified due to their PIT tag barcodes, with their offspring constituting a family. Families were obtained by semi-artificial breeding, implying that the fertilized eggs were taken from the mouth of the female and then transferred to a McDonald jar where they were incubated in a thermo-regulated system at 27 ± 2 °C for ∼8 days. They were then placed in 100L aquarium with re-circulating systems and reared at 27 ± 2 °C until the time of sexing. Temperature was regulated around 27 °C, which is the standard rearing temperature for this species and it does not influence sex (Baroiller et al., 1995). Progenies (G1 individuals) were fed *ad libitum* with commercial pellets (Skretting, Norway). They were exposed to 12L:12N photoperiod; temperature, pH and dissolved oxygen were checked two or three times per day during artificial incubation (0 to ∼8 days post-fertilization, dpf), sex differentiation (9 to 40 dpf) and during the growing phase until sexing at 90 dpf (Tables S2 and S4). A fragment of the caudal fin was sampled from each sacrificed fish and stored in absolute ethanol (99%) in order to analyze their genotype using the sex-linked markers.

**Figure 2:**
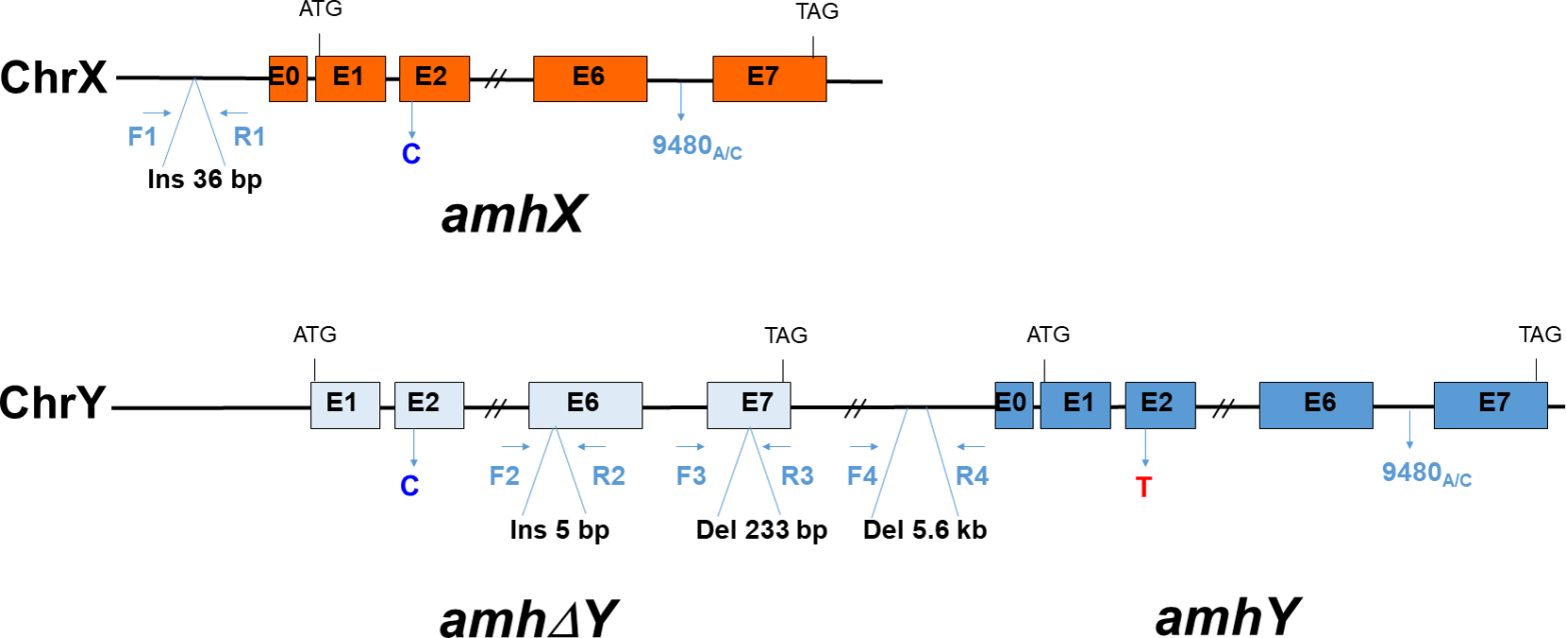
Schematic representation of the *amh*/*amh*ΔY/amhY gene structures on the Y and X chromosome of LG23, showing the location of some sex-linked markers (modified from Li et al., 2015). The coding sequence of *amhY* was identical to *amhX* except for a missense SNP (C/T) in exon 2 and a 5,608 bp deletion in the promoter. The *amhX* gene has insertions in the promoter region. The *amh*Δ*Y* gene has several insertions and deletions including a 5 bp insertion in exon 6 which induces a premature stop codon and subsequently, a truncated protein. The primer couples (arrows) used in the present study for the four *amh* sex-markers are shown and the corresponding inserted or deleted bp indicated. The SNP amh9480 is also shown.

To normalize sex ratios, around 100 randomly picked individuals were sexed for each family. However, power cuts occurred during the growing phase of some progenies eventually causing mortalities. All survivors were sexed when the number was below 100 (families Ko3, Ko6 and Ko7). In the Ko6 family, only 50 out of 56 individuals were sacrificed in order to keep 6 fish alive for further analysis. Sexing was performed at 90 dpf by gonadal squash, squashing a small portion of the gonad between a slide and coverslip, and then observing it under the microscope (Baroiller & D’Cotta, 2018). In order to eliminate the hypothesis of sex-linked mortalities, two repeated crossings were performed using the same parents of three couples to verify the stability of the sex-ratios. We have also induced mortalities by simulating rearing problems (power cuts and subsequent hypoxia) during the growing phase and sexed each fish upon their death, to verify that there was no sex-specific mortalities.

### Genomic DNA extraction

Genomic DNA (gDNA) was extracted from all tagged breeders (*n* = 91) and from some G1 individuals from the progeny testings families, obtained from crossing wild-caught parents. Extraction was performed following the protocol described by Khanam et al. (2016) using a small portion of the caudal fin (0.5 ×0.5 cm). Briefly, fin clips were digested at 55 °C overnight in lysis solution (0.3 M NaCl, 50 mM Tris-Base, 0.2 Mm EDTA, 0.2 mM EGTA, 0.356 mM spermidine, 0.256 mM spermine, 4.8% SDS) containing 30 µg proteinase K, followed by a 10µg RNAse treatment for 1 hr at 37 °C. Proteins were precipitated by adding 5M NaCl buffer and gDNA was isolated using 100% Isopropanol, washed in 70% ethanol and finally resuspended in 5 mM Tris buffer. The gDNA was quantified on the Qubit fluorimeter using the dsDNA BR kit (Qubit 2.0; Invitrogen), diluted to 30 ng/µl and stored at −20 °C until PCR reactions. In addition, the DNA quality was validated on 0.8% agarose gels and/or by nanodrop measurements.

**Table 1:** Sex markers used for the genotyping showing their polymorphism, primers sequences, amplification product and Chr specificity. Original marker name and corresponding references are indicated.

**Markers ID**	**Polymorphism detected**	**Primers sequences 5′–3′**		**Amplified fragments**	**Specificity for Chr X or Y (*amh*gene)**		**Product name & References**
**amhX**_+36_	36 bp insertion in *amhX* promoter	F1-	GTTTGCAATAGTTAGGGTGCTGCTG	1,000 bp	X	(*amhX*)	**Ins1**
		R1-	GGAAATGCAGCCATTCCTGAG				Li et al. (2015)
**amh**Δ**Y**_+5_	5 bp insertion in *amh*Δ*Y* Exon 6	F2-	AAACCTCCTTCCTTTGTGAATGTC	1,500 bp	Y	(*amh*Δ*Y*)	**Ins2**
		R2-	CTAGCGGCATCCACACTCCCTCAC				Li et al. (2015)
**amh**Δ**Y**_−233_	233 bp deletion in *amh*Δ*Y* Exon 6	F3-	CGGTCCCAGTGACCTATGAG	1,000 bp	X & Y	(*amhX; amhY*)	Eshel et al. (2014)
		R3-	AAGTACACGTGGTGTATTGTAATTGA	767 bp	Y	(*amh*Δ*Y*)	
**amhY**_−5608_	5608 bp deletion in *amhY* promoter	F4-	GAAAGGGGTGTTTTGGTGCTGGC	8,022 bp	X	(*amhX*)	**Del5**
		R4-	ACCCAGGAAGCGTTTCATCTCA	2,414 bp	Y	(*amhY*)	Li et al. (2015)

### Genotyping with X and Y chromosome markers

The two *amh* gametologs and *amh* Δ*Y* (Fig. 2) identified on LG23 in a Japanese strain are distinguishable by several insertions and deletions located in the promoter and in exons 6 and 7 (Li et al., 2015). Primers designed by Li et al. (2015) for these regions allowed them to differentiate XX, XY and YY genotypes in their strain. In the present study we used a combination of 4 markers specific for the X and/or for the Y chromosomes (Table 1). The amhX _+36_ (=Ins1 of Li et al., 2015) marker is specific for the *amhX* gametolog, amplifying a 1,000 bp fragment in both males and females carrying an X chromosome. The presence of the *amh* Δ*Y* gene was ascertained by two markers. One was the amh ΔY_+5_ (=Ins2 of Li et al., 2015) marker with the amplification of the 5 bp insertion which changes the reading frame. The second was the amh ΔY_−233_ marker which amplifies when present an *amh* Δ*Y* -specific fragment of ∼767 corresponding to a 233 bp deletion, and amplifies a non-specific fragment of ∼1,000 bp fragment corresponding to both the *amhX* and *amhY* gametologs (Eshel et al., 2014). The marker named amhY_−5608_ (=Del5 of Li et al., 2015) is associated to both the *amhX* (8022 bp fragment) and the 5,608 bp deleted promoter fragment of the *amhY* (2,414 bp fragment) indicating respectively the presence of X and Y chromosome. The PCR reactions for amhX_+36_, amh ΔY_+5_ and amh ΔY_−233_ were performed in 25µl using 3 µl of DNA (30 ng/µl) with a normal Taq polymerase (MP Biomedicals) whereas for the amhY_-5608_ marker, because of the size of the amplified fragments (8,022 bp and 2,414 bp), amplification was performed with the LongAmp Taq (New England Biolabs) using 4.5 µl of DNA (30 ng/µl) in a 25µl PCR reaction. The PCR program for amhX_+36_ was an initial denaturation at 94 °C for 3 min, followed by 36 cycles of amplification at 94 °C for 30s, 62 °C for 45s and 72 °C for 2min, and then a final elongation step (72 °C for 10min). The program for amh ΔY_+5_ was a denaturation at 94 °C for 3 min, followed by a touchdown of 10 cycles consisting in 94 °C for 30s, annealing from 68 to 64 °C for 45s and 72 °C for 2min, and 26 cycles at Tm of 64 °C, and final elongation at 72 °C for 10min. For the amh ΔY_-233_ the program was 94 °C for 3 min, 38 cycles of amplification at 94 °C for 30 s, 58 °C for 45s and 72 °C for 1min 30s, and an elongation at 72 °C for 10min. For the amhY_-5608_ the program was 94 °C for 20 s, followed by 38 cycles of amplification at 94 °C for 30s, 60 °C for 30s and 65 °C for 7min, and an elongation step at 65 °C for 10min. PCR products were separated on a 1.5% agarose gel for all markers except the amhY_-5608_ products for which 1% agarose gel was used in order to facilitate the migration of large fragments.

### Kompetitive allele specific PCR (KASP^TM^)

Genotyping using the KASP assay was performed for an SNP OniAmh9480 located in intron 6 (LG23:9603363) of the *amh* gene (Table S1). Although this SNP is located in a non-coding region, it was strongly associated to the phenotypic sex in the Lake Kpandu population from Ghana (Table S5) which shares the same Volta basin as Lake Kou, draining into the Volta River. In addition, the supposedly *amhY* diagnostic missense SNP C/T present in exon 2 (Li et al., 2015) and the exon 6 missense G/C (rs431905480; (Wessels et al., 2014) were tested. Two supposedly diagnostic loci for LG1 Oni23063, intron variant A/G (rs397507167) and Oni28137, intron variant (rs397507165) (Palaiokostas et al., 2013) were also tested (Table S6).

KASP is a fluorescent genotyping assay based on competitive allele-specific PCRs which allows bi-allelic scoring of SNPs at specific loci. Forward FAM and HEX primers and common reverse primers designed by KBiosciences are shown in Table S4. PCR reactions were performed on the LightCyclerR480 (Roche) in 5 µl reaction mixture consisting in 2 µL DNA (10 ng/ µL), 2 µL KASP master mix, 0.055 µL KASP assay primers and 0.032 µL MgCl_2_. The thermal cycling condition were: initial denaturation at 94 °C for 15 min, followed by 10 cycles at 94 °C for 20 s, touchdown over 65 °C to 57 °C for 1 min, and 28 cycles at 94 °C for 20 s and 57 °C for 1min.

### Data analysis

The expected and observed amhs’ genotypes were compared using the Fisher’s exact test (OpenEpi 3.01 Software). The observed and expected distributions of the phenotypes and the assigned genotypes were analysed using a binomial test (Minitab^®^14 software). Data clustering analysis of the KASP assay was performed using the endpoint genotyping method (LightCycler^®^480 1.5 software). The FAM and HEX fluorescence were detected at 483–533 nm and 523–568 nm respectively. For each individual the fluorescence values indicated the presence of allele FAM (A) and/or allele HEX (C). The SNP genotypes were analyzed using Fisher’s exact test. Sex ratios were compared to ratios 1:1 (MXY  × FXX), to 0:1 (MXX  × FXX) and 3:1(MXY  × FXY) due to the probability of sex-reversal (male-to-female or female-to-male) and were analysed statistically using the Fisher’s exact test. For statistical analyses, the level of significance was accepted at *P* < 0.05; the significance at *P* < 0.01 and *P* < 0.001 are also shown. A continuity correction was applied when Binomial test and Fisher’s exact test included zero values. Fisher’s exact test was used to compare the sex ratios between two repeated crossings in three families and with a balanced 1:1 sex ratio. It was also used to compare the sex of dead fish in the induced mortality test *versus* the observed sex-ratio. To validate the progeny testing data, we assessed the correlation between the stocking density, mortality rate, the number of fish sexed and the sex-ratio (Tables S7; S8), using Pearson correlation test.

## Results

### Validation of the genotypic sexing on two domestic strains using *amh* X and Y markers

**Figure 3:**
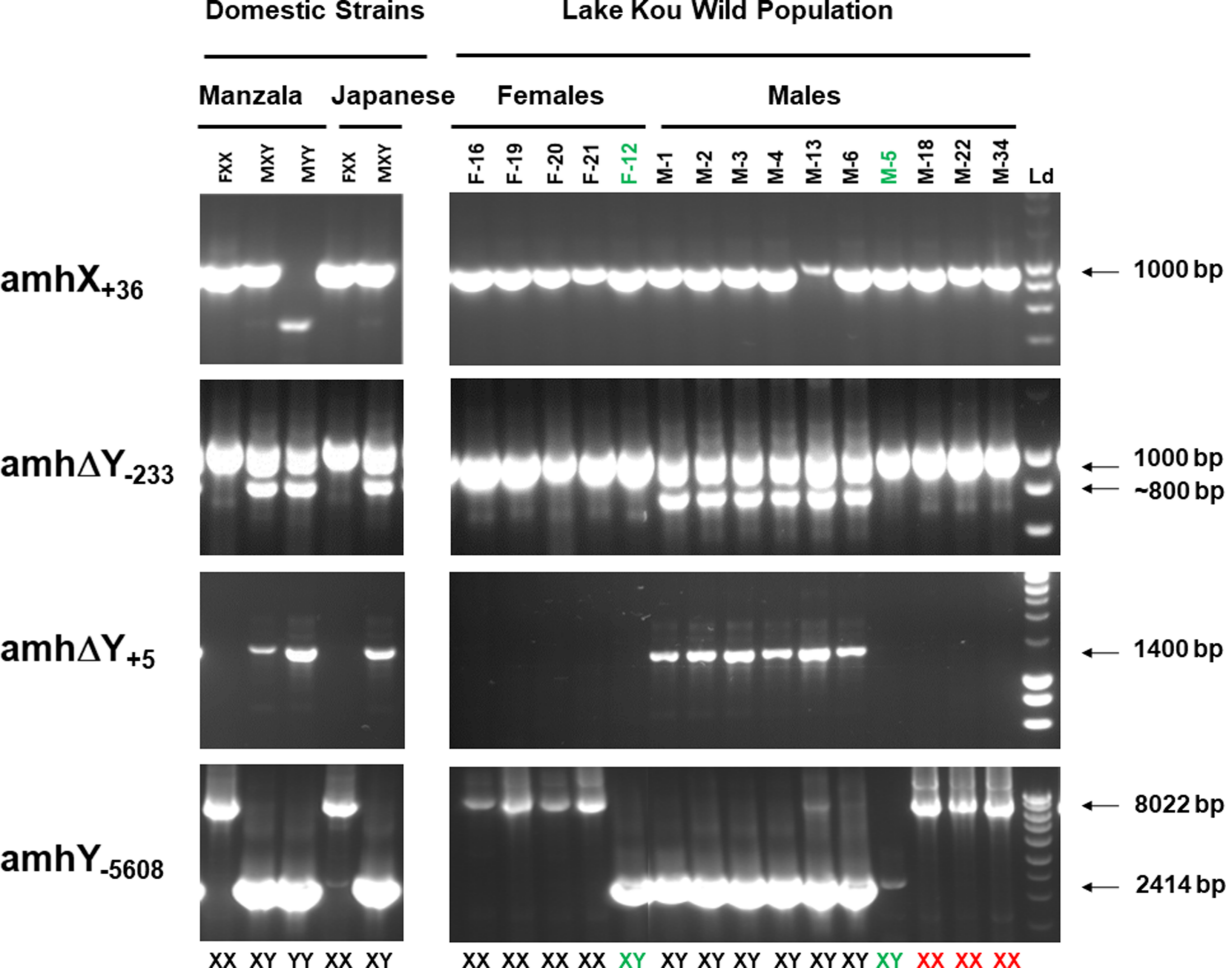
Genetic sex assignment using four *amh* markers of males and females from two domestic strains (Manzala and Japanese) of Nile tilapia and of wild fish from Lake Kou. The markers indicate the presence of the *amhX*, *amh*Δ*Y* and *amhY* genes which are correlated to X and Y chromosomes in the LG23 system. The 1,000 bp amplification with *amhX*_+36_ is X chromosome specific. *Amh*Δ*Y*_−233_ is associated with a 1,000 bp for X and Y amplification and a ∼800 bp for the Y chromosome, *amh*Δ*Y*_+5_ is Y specific and *amhY*_−5608_ is X (8,022 bp) and Y (2,414 bp) chromosome specific. The Japanese strain was used to validate the genotyping procedure, showing amplifications found on an XX female and an XY male. From the Manzala strain different known genotypes consisting in female XX, and XY and YY males were used. Males and females captured in Lake Kou were also analyzed and gel image shows amplifications with the four amh markers on some of the individuals analyzed, indicating XX females and XY males with putative XX males (in red). The particular XY female is indicated as F12 and the XY male M5 genotyped as *amh* (*X*^+^Δ*Y*^−^*Y*^+^) are shown in green. Ld = DNA ladder.

We first verified the amplifications of the four markers on males and females of the Japanese strain, used by Li et al. (2015) for the *amhs* analyses. Markers were subsequently validated in the Manzala strain on known sex genotypes. In both strains, females lacked the *amh* Δ*Y* gene as seen by the absence of amplification with the amh ΔY_+5_ marker as well as the absence of a 767 bp fragment for the amh ΔY_-233_ marker (Fig. 3). This amh ΔY_−233_ marker also amplifies a higher ∼1,000 bp band which can correspond to the *amhX* and/or the *amhY* genes. Presence of the amhX_+36_ 1,000 bp fragment indicates the existence of an X chromosome. The amhY_-5608_ marker is also specific to the *amhX* gene amplifying a 8,022 bp fragment in the promoter region, but this fragment is not always amplified when individuals carry both X and Y chromosomes, with preferential amplification when present, of the *amhY* 2,414 bp band (Fig. 3). We used this marker mostly to determine the presence of the *amhY* gene which is specific for this 2,414 bp fragment. A very slight band was observed in a Japanese female but this female was considered an XX giving balanced 1:1 sex ratios when it was tested with XY males. Manzala XY and YY males both amplified strongly the 2,414 bp fragment corresponding to the *amhY* gene with the amhY_-5608_ marker although the 8 kb band could not be observed in the XY males. In YY males the amhX_+36_ 1,000 bp fragment corresponding to the *amhX* gene was absent but interestingly a 600 bp band was observed and this was consistent to several YY individuals. Therefore, our genotypic results matched perfectly the known sex genotypes of the Manzala (obtained from several previous hormonal treatments followed by progeny testings) and the Japanese fish (Table 2). Regardless of the phenotypic sex, the corresponding *amh* genotype was *amh* (*X*^+^Δ*Y*^−^*Y*^−^) for XX individuals, *amh* (*X*^+^Δ*Y*^+^*Y*^+^) for XY individuals and *amh* (*X*^−^Δ*Y*^+^*Y*^+^) for YY individuals.

**Table 2:** Sex chromosome LG23 markers analysed in two domestic strains. Genotype assigned using the four amh markers, match the known genotype.

**Domestic strains**	**Phenotype**	**N analyzed**	**Genotype**	***amhX*genotype**	***amh***Δ***Y*genotype**	***amhY*genotype**	***amhs*genotype**	**Correspondence of ratio**
**Japanese**	**Males**	2	**XY**	*amhX*^+^	*amh*Δ*Y*^+^	*amhY*^+^	**XY**	1
** **	**Females**	2	**XX**	*amhX*^+^	*amh*Δ*Y*^−^	*amhY*^−^	**XX**	1
**Manzala**	**Males**	2	**XX**	*amhX*^+^	*amh*Δ*Y*^−^	*amhY*^−^	**XX**	1
** **		3	**XY**	*amhX*^+^	*amh*Δ*Y*^+^	*amhY*^+^	**XY**	1
** **		3	**YY**	*amhX*^−^	*amh*Δ*Y*^+^	*amhY*^+^	**YY**	1
** **	**Females**	3	**XX**	*amhX*^+^	*amh*Δ*Y*^−^	*amhY*^−^	**XX**	1
** **		2	**XY**	*amhX*^+^	*amh*Δ*Y*^+^	*amhY*^+^	**XY**	1
** **		2	**YY**	*amhX*^−^	*amh*Δ*Y*^+^	*amhY*^+^	**YY**	1

### Genotypic sexing of wild caught fish using *amh* X and Y markers

In the Lake Kou population, our analysis revealed that all males and females were *amhX*^+^, suggesting the presence of an X chromosome. All females were *amh* Δ*Y*^−^ and surprisingly, 15 out of 46 males were also *amh* Δ*Y*^−^ (*P* = 0.00016). Therefore, the truncated gene was present in only 67.39% males. In all the females, *amhY* was not found except in one female for which we found the 2,414 bp band (Fig. 3). All the 15 males which were *amh* Δ*Y*^−^, were also *amhY*^−^, except one which was *amhY*^+^. These results suggest that the presence of the *amh* Δ*Y* gene is not always associated with the *amhY* gene. Taken together, the results show a significant proportion of mismatches between genetic and phenotypic sex mostly in male individuals (Table 3). In males we expected 100% for each gene and these ratios should have been 100%, 0% and 0%, respectively, for *amhX, amh* Δ*Y* and *amhY* in females. No significant (*P* > 0.99999) differences in *amhY* ratio (1/45) were observed in females. In contrast, males presented significant differences (*P* = 0.00016 and *P* = 0.00036) in *amh*  Δ *Y* (31/46) and *amhY* ratios (32/46) respectively.

**Table 3:** Genetic sex assignment of Lake Kou individuals using 4 Amh sex chromosome markers. Observed vs. Expected numbers were compared using the Fisher exact test. For 0 values, a continuity correction was applied (by adding 0.5 to each value).

**Phenotype**	**Genotype assigned**	***amhX*genotype**	***amh***Δ***Y*genotype**	***amhY*genotype**	**Observed N**	**Expected N (1:1)**	***P*value**	**Sig.**
Males	**XY**	*amhX*^+^	*amh*Δ*Y*^+^	*amhY*^+^	31	46	0.00016	^***^
	**XY**	*amhX*^+^	*amh*Δ*Y*^−^	*amhY*^+^	1	0	>0.99999	NS
	**XX**	*amhX*^+^	*amh*Δ*Y*^−^	*amhY*^−^	14	0	0.00036	^***^
Females	**XX**	*amhX*^+^	*amh*Δ*Y*^−^	*amhY*^−^	44	45	>0.99999	NS
	**XY**	*amhX*^+^	*amh*Δ*Y*^−^	*amhY*^+^	1	0		

**Notes.**

Significance (Sig): *5%; **1%; ***0.1% and NS for non-significant.

**Figure 4:**
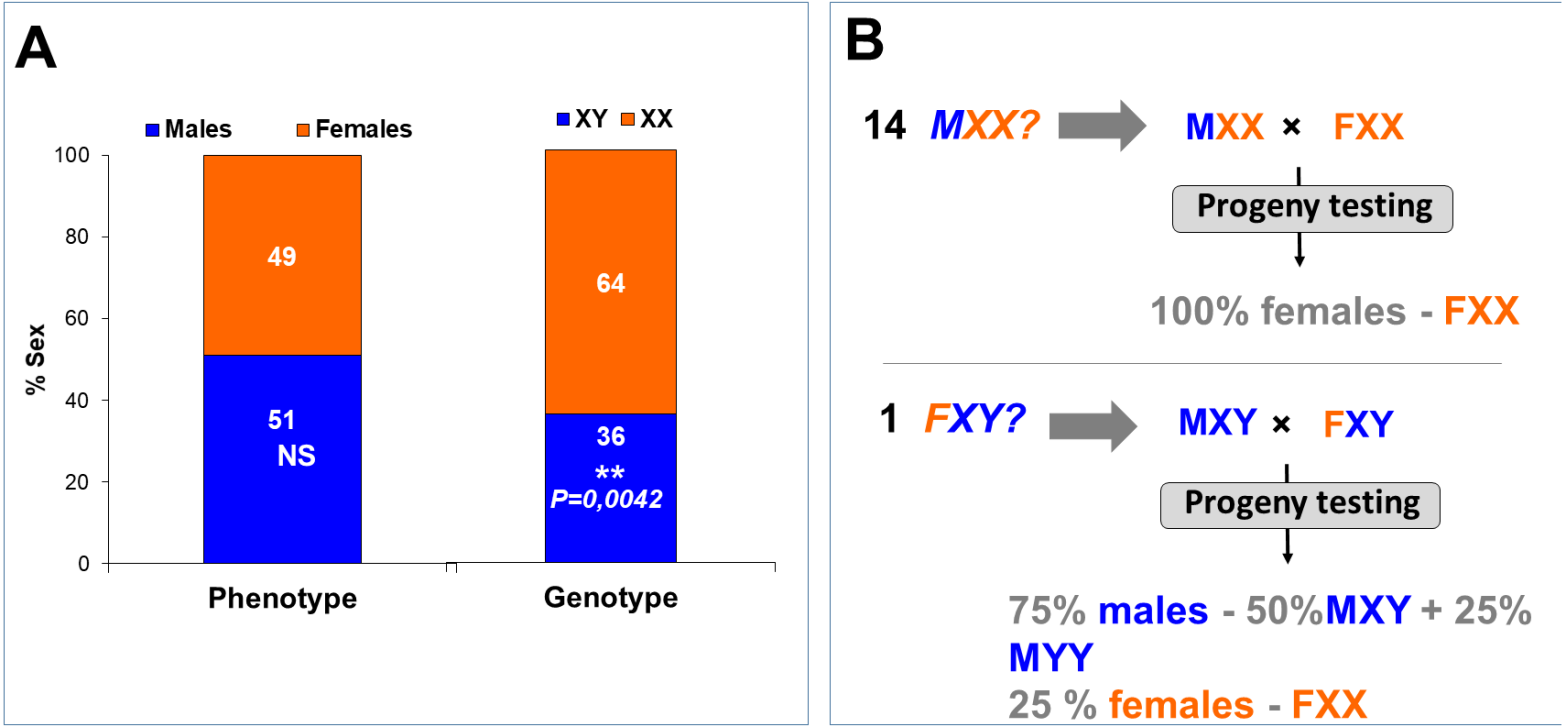
Proportions of males and females from the wild Kou fish and the assigned XX and XY genotypes. (A) Proportions of male and female phenotypes identified in 91 wild-caught tilapia from the Kou Lake, and the genotypes assigned with the *amh* sex-markers, consisting in 14 putative XX males and one XY female. Females are shown in orange and males in blue. (B) Progeny testing of an XX male is expected to give a sex-ratio of 0:1 (100% females). The progeny testing of the XY female is expected to give a sex-ratio of 3:1 (75% males of which 25% are YY males, plus 25% of XX females.

According to the presence or absence of the three *amhs* genes or copies, we genotyped in the captured Kou fish 67.39% of the males as *amh* (*X*^+^Δ*Y*^+^*Y*^+^) which were assigned as XY males whereas, 97.78% of the females with *amh* (*X*^+^Δ*Y*^−^*Y*^−^) genotype were assigned as XX females. Some males (30.43% of males) were genotyped as *amh* (*X*^+^Δ*Y*^−^*Y*^−^) and were assigned as putative sex reversed XX males. In addition, the *amh* (*X*^+^Δ*Y*^−^*Y*^+^) genotype was observed in a low proportion (*n* = 1) of males (2.17%) and females (2.22%). These new profiles were not found in domestic strains previously analyzed. Consequently, the female with *amh* (*X*^+^Δ*Y*^−^*Y*^+^) genotype could be a XY female. To summarize, the phenotypic sex was balanced (*P* = 0.9409) in the captured Kou fish but the genotypic sex appears to be significantly biased towards an XX genotype (*P* = 0.0042) (Table 3; Fig. 4A). According to the *amh* X and Y chromosome markers, we found five phenotype/genotype combinations consisting in 31 XY-*amh* (*X*^+^Δ*Y*^+^*Y*^+^) males, 44 XX-*amh* (*X*^+^Δ*Y*^−^*Y*^−^) females, 14 XX-*amh* (*X*^+^Δ*Y*^−^*Y*^−^) males, one XY- *amh* (*X*^+^Δ*Y*^−^*Y*^+^) male and one XY- *amh* (*X*^+^Δ*Y*^−^*Y*^+^) female.

### Sex linkage of the SNP OniAmh9480 in Lake Kou population

**Figure 5:**
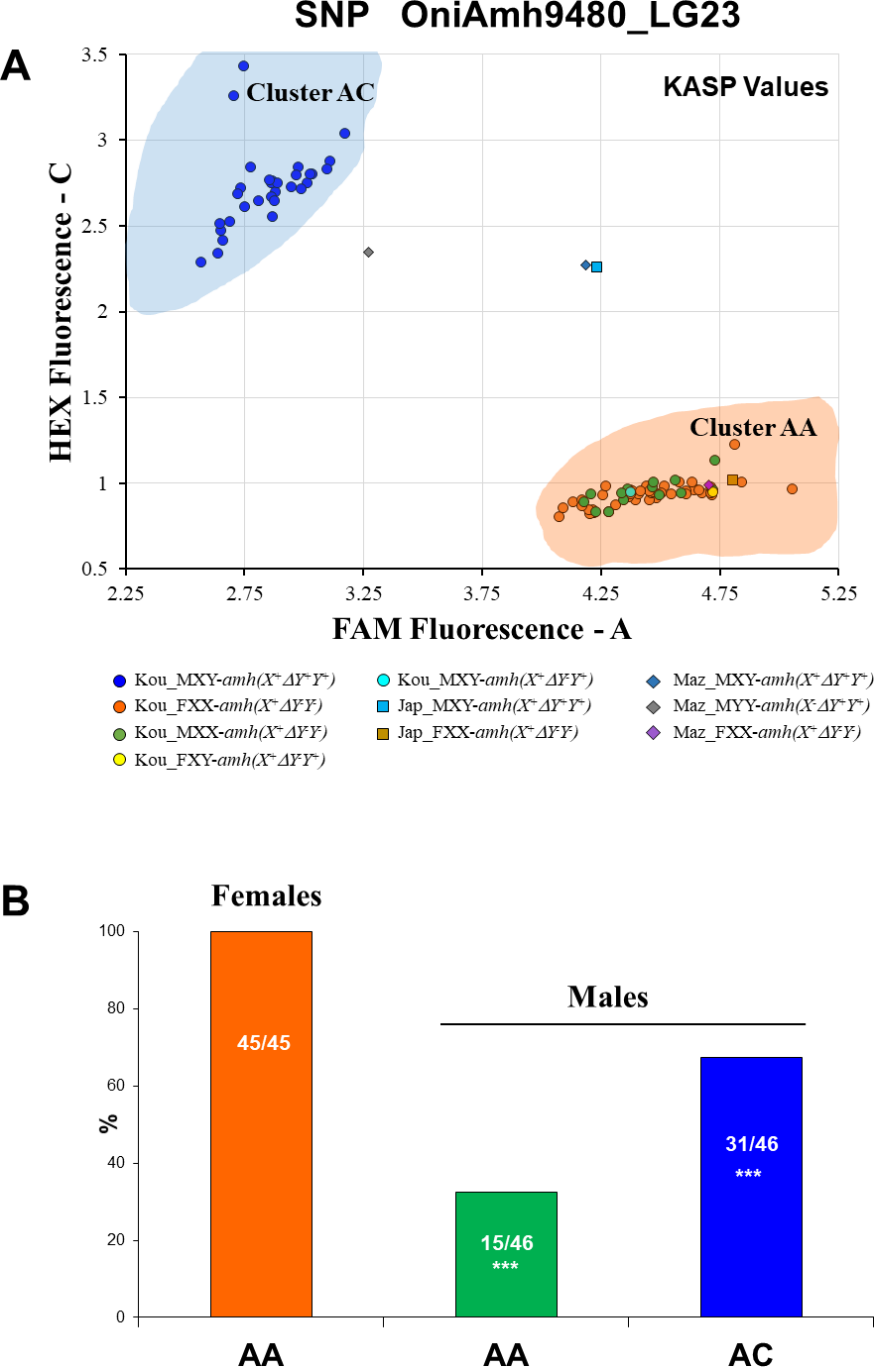
KASP assay of the SNP OniAmh9480. (**A**) Fluorescence values obtained for each fish from the Kou population. XY Males with *amh*(*X*^+^Δ*Y*^+^*Y*^+^) genotype were grouped in a heterozygous cluster AC (blue circles). All the individuals which were *amh*Δ*Y*^−^, including the putative FXX (orange circles), MXX (green circles) genotyped as *amh*(*X*^+^Δ*Y*^−^*Y*^−^), the particular FXY (yellow circle) and MXY (aqua circles) genotyped as *amh*(*X*^+^Δ*Y*^−^*Y*^+^), were located in the homozygous cluster AA. Both the Manzala (Maz) female (purple diamond) and Japanese (Jap) female (brown square) clustered with the Kou females. In contrast both the Manzala and Japanese XY males as well as the Manzala YY male, were positioned outside both clusters, towards the center. (B) Proportions of the alleles identified by KASP, indicating the number of alleles observed per phenotypic sex, with 45 females out of 45 being homozygous AA (orange), 15 out of 46 males were homozygous AA individuals (green) while only 31 males were found to be AC heterozygotes (blue).

We used a KASP assay to study the sex association to 5 SNPs of the Kou fish. Tests showed that only the SNP OniAmh9480 (A/C) was polymorphic in the Kou population. This SNP data clearly clustered the 91 fish into 2 groups consisting in homozygous AA and heterozygous AC genotypes (Fig. 5A). All females (*n* = 45) were homozygous AA. The majority of males were heterozygous AC (67.4%), but 15 males out of 46 (32.6%) did not have the nucleotide transition and were located in the homozygous cluster (Fig. 5). The deviation from the expected number is highly significant (*P* < 0.000007). These proportions revealed that the AA and AC genotypes could be respectively genetic XX and XY individuals. Combined data analysis of SNP genotypes and *amh*-assigned genotypes (Table 4; Table S9), showed that all males which displayed the *amh* (*X*^+^Δ*Y*^+^*Y*^+^) genotype were also AC heterozygotes for the OniAmh9480 marker. All assigned XX males with *amh* (*X*^+^Δ*Y*^−^*Y*^−^) genotype were AA homozygotes and the XX females with *amh* (*X*^+^Δ*Y*^−^*Y*^−^) were likewise, AA homozygotes. Surprisingly, the male and female which had *amhY* without *amh* Δ*Y* were AA homozygotes, suggesting an XX genotype instead of an XY genotype. Both Japanese and Manzala males were found to be AC heterozygotes clustering separately from the Kou XY males. The YY Manzala male was not a CC homozygote but rather an AC heterozygote clustering with the XY males from Kou.

**Table 4:** Genotypic discrimination using SNP OniAmh9480 of Lake Kou Nile tilapia. Observed vs. Expected numbers were compared using the Fisher exact test. For 0 values, a continuity correction was applied (by adding 0.5 to each value). Alleles frequencies were calculated as follow: *f*(*A*) = (2 × *NAA* + *NAC*)∕2*Nt* and *f*(*C*) = *NAC*∕2*Nt* with NAA and NAC representing the number of individuals genotyped as AA and AC respectively; Nt, total number of males and females.

Phenotype	SNP Genotype	Observed N	Expected N	*P* value	Sig.	Genotype frequency	Alleles frequencies Obs.	Alleles frequencies Exp.	Genotype assigned	Amh genotypes
Males	AC	31	46	<0.000007	^***^	0.341	0.83	0.747	XY	*amh(X*^+^Δ*Y*^+^*Y*^+^*)*
	AA	15	0			0.659	0.17	0.253	XX	*amh(X*^+^Δ*Y*^−^*Y*^−^*)*
									XY	*amh(X*^+^Δ*Y*^−^*Y*^+^*)*
Females	AA	45	45	>0.9999999	NS				XX	*amh(X*^+^Δ*Y*^−^*Y*^−^*)*
									XY	*amh(X*^+^Δ*Y*^−^*Y*^+^*)*

**Notes.**

Significance (Sig): *5%; **1%; ***0.1% and NS for non-significant.

### Progeny testings of putative XX males and an XY female

We assigned 14 fish as XX males having the *amh* (*X*^+^Δ*Y*^−^*Y*^−^) genotype. To verify their genotypes, we crossed eight of these males with different wild caught females, identified as being normal genetic XX females since they possessed the *amh* (*X*^+^Δ*Y*^−^*Y*^−^) genotype (Fig. 4B). XX males crossed with XX females give theoretically 100% XX females (**0:1** sex ratio; Fig. 4B). Offspring of seven of these males showed instead a balanced **1:1** male to female sex ratios (Table 5). One assigned XX male (M61) however gave 100% females for family Ko11 (Sex ratio **1:1**–*P* < 0.0000001^∗∗∗^; Sex ratio **0:1**–*P* >0.9999999^**NS**^). Because sex ratios in Nile tilapia are influenced by minor genetic factors brought by both parents, we further crossed some of the putative XX males with other assigned genetic XX females. Male M72 sired a balanced 1:1 sex ratio for Ko10 (*P* = 0.8876^**NS**^) and likewise, male M68 for Ko22 also gave a balanced sex ratio (*P* > 0.9999999^**NS**^). The M61 XX male gave 99% females in the Ko15 family (Sex ratio **1:1** *P* < 0.0000001^∗∗∗^; Sex ratio **0:1**–*P* > 0.9999999 ^**NS**^), which confirmed that it was indeed an XX male.

**Table 5:** Progeny testings of different wild Kou fish showing the comparative analysis of the expected 1:1 sex ratios versus the observed using Fisher exact test.

**Families**	**Crossings of Male & Female breeders**	**Breeders ID**	**Mortality rate %**	**N sexed**	**Sex ratio (% males)**	**Fisher exact test**	
						***P***	**Sig.**
**Ko3**	**MXY-***amh(X*^+^Δ*Y*^+^*Y*^+^) × **FXX-***amh(X*^+^Δ*Y*^−^*Y*^−^)	M- 89 × F-57	74	47	51	>0.9999999	***NS***
**Ko7**		M- 3 × F-94	60	25	44	0.6713	***NS***
**Ko19**		m- 109 × F-28	17	100	52	0.8876	***NS***
**Ko21**		m- 109 × F-100	0	48	54	0.8383	***NS***
**Ko31**		M- 116 × F-36	11	100	44	0.4788	***NS***
**Ko32**		M- 117 × F-25	18	100	51	>0.9999999	***NS***
**Ko5**	**MXX-***amh(X*^+^Δ*Y*^−^*Y*^−^) × **FXX -***amh(X*^+^Δ*Y*^−^*Y*^−^)	M- 34 × F-75	42	100	45	0.5712	***NS***
**Ko8**		M- 80 × f-82	38	100	46	0.6712	***NS***
**Ko9**		M- 72 × F-75	17	100	52	0.8876	***NS***
**Ko10**		M- 72 × F-78	23	100	52	0.8876	***NS***
**Ko11**		M- 61 × F-112	20	109	0	<0.0000001	^***^
**Ko13**		M- 18 × F-121	22	111	48	0.7372	***NS***
**Ko14**		M- 84 × F-75	28	99	47	0.7224	***NS***
**Ko15**		M- 61 × F-58	33	106	1	<0.0000001	^***^
**Ko18**		M- 68 × F-25	28	108	52	0.8918	***NS***
**Ko22**		M- 68 × F-124	14	100	50	>0.9999999	***NS***
**Ko25**		M- 37 × F-75	48	100	48	0.8876	***NS***
**Ko6**	**MXY-** amh(X^+^Δ*Y*^−^*Y*^+^) × **FXX-***amh(X*^+^Δ*Y*^−^*Y*^−^)	M- 5 × F-57	5	50	0	<0.0000001	^***^
**Ko12**		M- 5 × F-25	31	100	48	0.8876	***NS***
**Ko17**	**MXY-***amh(X*^+^Δ*Y*^+^*Y*^+^) × **FXY***amh(X*^+^Δ*Y*^−^*Y*^+^)	M- 50 × F-12	19	100	61	0.1546	***NS***
**Ko29**		M- 50 × F-12	19	102	57	0.3998	***NS***
**Ko24**		M- 71 × F-12	14	102	70	0.006499	^**^

**Notes.**

Significance (Sig): *5%; **1%; ***0.1% and NS for non-significant

We also crossed the male M5 which had the *amh* (*X*^+^Δ*Y*^−^*Y*^+^) genotype. This male sired a 100% female progeny when crossed with the female F57 (Ko6 family) (Sex ratio **1:1**–*P* < 0.0000001^∗∗∗^) which suggests it is an XX male. However, a subsequent crossing with another female F25 resulted in a balanced sex-ratio (Ko12) (Sex ratio **1:1**–*P* = 0.8876^**NS**^) which suggests instead that M5 might be an XY male.

The female assigned as XY due to her *amh* (*X*^+^Δ*Y*^−^*Y*^+^) genotype was crossed three times with two XY males. When crossed with male M50 it gave more or less balanced sex ratios of 61% and 58% males (Table 5). However, when crossed with another XY male (M71) it gave 70% males which corresponds to the expected **3:1** sex ratio (*P* = 0.63^**NS**^) of a putative XY female (Fig. 4B).

To further test the genotype assignments, we also crossed G0 males which were assigned as XY males having the *amh* (*X*^+^Δ*Y*^+^*Y*^+^) genotype. These males all sired offspring with balanced sex-ratios (families Ko3, Ko7, Ko19, Ko21, Ko31 and Ko32) (Table 5). In order to normalize sex ratios per family, around 100 individuals were sexed but for some families it had to be done on 25 fish and for others on 111 fish. Our testing showed that this variation did not induce significant differences in the sex ratios (Table 6). Likewise, sex ratio were not significantly correlated with stocking density and/or survival rate (Table 6).

**Table 6:** Correlations between the stocking density, mortality rates, male ratios and the number of fish sexed analyzed with the Pearson correlation test.

	**Stocking density**		**Mortality rate**		**Sex ratio (% males)**	
	**Rho-value**	***P*-value**	**Rho-value**	***P*-value**	**Rho-value**	***P*-value**
**Stocking density**	** **	* *				
**Mortality rate**	0.364	0.096^NS^	* *	* *		
**Sex ratio (% males)**	0.133	0.554^NS^	0.043	0.849^NS^	* *	* *
**N fishes sexed**					−0.279	0.208^NS^

**Notes.**

Rho-values represent the Pearson correlation coefficient.

Significance (Sig): *5%; **1%; ***0.1% and NS for non-significant.

**Table 7:** Amh genotype assignment of G1 individuals using 4 Amh sex chromosome markers, showing the number of individuals for each possible genotype and the proportion per sex observed.

**Crossings of wild male & female breeders**	**Families**	**Breeders ID**	**Sex ratio (% males)**	**Phenotype**	**Amh genotype**	**Observed N/ Total N**	**% per sex**
**MXY***amh(X*^+^Δ*Y*^+^*Y*^+^) × **FXX***amh(X*^+^Δ*Y*^−^*Y*^−^)	**Ko19**	m- 109 × F-28	52	Males	*amh(X*^+^Δ*Y*^+^*Y*^+^*)*	30/30	100
					*amh(X*^+^Δ*Y*^−^*Y*^+^*)*	0/30	0
					*amh(X*^+^Δ*Y*^−^*Y*^−^*)*	0/30	0
				Females	*amh(X*^+^Δ*Y*^+^*Y*^+^*)*	1/30	3
					*amh(X*^+^Δ*Y*^−^*Y*^+^*)*	0/30	0
	** **	** **	** **		*amh(X*^+^Δ*Y*^−^*Y*^−^*)*	29/30	97
**MXX***amh(X*^+^Δ*Y*^−^*Y*^−^) × **FXX***amh(X*^+^Δ*Y*^−^*Y*^−^)	**Ko15**	M- 61 × F-58	1	Females	*amh(X*^+^Δ*Y*^+^*Y*^+^*)*	0/28	0
					*amh(X*^+^Δ*Y*^−^*Y*^+^*)*	0/28	0
	** **	** **	** **		*amh(X*^+^Δ*Y*^−^*Y*^−^*)*	28/28	100
	**Ko22**	M- 68 × F-124	50	Males	*amh(X*^+^Δ*Y*^+^*Y*^+^*)*	0/30	0
					*amh(X*^+^Δ*Y*^−^*Y*^+^*)*	0/30	0
					*amh(X*^+^Δ*Y*^−^*Y*^−^*)*	30/30	100
				Females	*amh(X*^+^Δ*Y*^+^*Y*^+^*)*	0/30	0
					*amh(X*^+^Δ*Y*^−^*Y*^+^*)*	0/30	0
	** **	** **	** **		*amh(X*^+^Δ*Y*^−^*Y*^−^*)*	30/30	100
**MXY***amh(X*^+^Δ*Y*^−^*Y*^+^) × **FXX***amh(X*^+^Δ*Y*^−^*Y*^−^)	**Ko6**	M- 5 × F-57	0	Females	*amh(X*^+^Δ*Y*^+^*Y*^+^*)*	0/30	0
					*amh(X*^+^Δ*Y*^−^*Y*^+^*)*	0/30	0
	** **	** **	** **		*amh(X*^+^Δ*Y*^−^*Y*^−^*)*	30/30	100
	**Ko12**	M- 5 × F-25	48	Males	*amh(X*^+^Δ*Y*^+^*Y*^+^*)*	0/30	0
					*amh(X*^+^Δ*Y*^−^*Y*^+^*)*	2/30	7
					*amh(X*^+^Δ*Y*^−^*Y*^−^*)*	28/30	93
				Females	*amh(X*^+^Δ*Y*^+^*Y*^+^*)*	0/30	0
					*amh(X*^+^Δ*Y*^−^*Y*^+^*)*	2/30	7
	** **	** **	** **		*amh(X*^+^Δ*Y*^−^*Y*^−^*)*	28/30	93
**MXY***amh(X*^+^Δ*Y*^+^*Y*^+^) × **FXY***amh(X*^+^Δ*Y*^−^*Y*^+^)	**Ko17**	M- 50 × F-12	61	Males	*amh(X*^+^Δ*Y*^+^*Y*^+^*)*	28/29	97
					*amh(X*^+^Δ*Y*^−^*Y*^+^*)*	1/29	3
					*amh(X*^+^Δ*Y*^−^*Y*^−^*)*	0/29	0
				Females	*amh(X*^+^Δ*Y*^+^*Y*^+^*)*	0/30	0
					*amh(X*^+^Δ*Y*^−^*Y*^+^*)*	19/30	63
	** **	** **	** **		*amh(X*^+^Δ*Y*^−^*Y*^−^*)*	11/30	37
	**Ko29**	M- 50 × F-12	57	Males	*amh(X*^+^Δ*Y*^+^*Y*^+^*)*	49/51	96
					*amh(X*^+^Δ*Y*^−^*Y*^+^*)*	2/51	4
					*amh(X*^+^Δ*Y*^−^*Y*^−^*)*	0/51	0
				Females	*amh(X*^+^Δ*Y*^+^*Y*^+^*)*	1/40	2.5
					*amh(X*^+^Δ*Y*^−^*Y*^+^*)*	26/40	65
	** **	** **	** **		*amh(X*^+^Δ*Y*^−^*Y*^−^*)*	13/40	32.5
	**Ko24**	M- 71 × F-12	70	Males	*amh(X*^+^Δ*Y*^+^*Y*^+^*)*	60/70	86
					*amh(X*^+^Δ*Y*^−^*Y*^+^*)*	10/70	14
					*amh(X*^+^Δ*Y*^−^*Y*^−^*)*	0/70	0
				Females	*amh(X*^+^Δ*Y*^+^*Y*^+^*)*	3/30	10
					*amh(X*^+^Δ*Y*^−^*Y*^+^*)*	17/30	57
	** **	** **	** **		*amh(X*^+^Δ*Y*^−^*Y*^−^*)*	10/30	33

Repeated crossings with the same Kou parents demonstrated in the present study that mortalities are not sex biased (Table S10). In addition, the simulation of a technical problem also proved that there was no sex-specific mortalities (Table S11).

### Genotypic sexing of G1 progeny-tested fish using *amh* X and Y markers

We performed genotype assignments with the amh markers on G1 male and female individuals from eight of the progeny tested families (Table 7; Table S12). Genotyping was done on 28 to 100 individuals. In the balanced Ko19 family, phenotypic males had the same *amh* (*X*^+^Δ*Y*^+^*Y*^+^) genotype as their father and likewise, the females had the *amh* (*X*^+^Δ*Y*^−^*Y*^−^) genotype of their mother. Surprisingly one female had the same genotype as her father. All the 28 females analyzed from the Ko15 family crossed with the identified XX male (M61) were *amh* (*X*^+^Δ*Y*^−^*Y*^−^). In contrast the M68 male despite also having an *amh* (*X*^+^Δ*Y*^−^*Y*^−^) genotype, sired a 50% male sex ratio in the Ko22 family where 30 males analyzed had all the *amh* (*X*^+^Δ*Y*^−^*Y*^−^) genotype as expected for XX males. In addition, the 30 females analyzed from this family had all female *amh* (*X*^+^Δ*Y*^−^*Y*^−^) genotypes.

The M5 male with a particular *amh* (*X*^+^Δ*Y*^−^*Y*^+^) genotype, sired the all-female Ko6 family which were all genotyped as *amh* (*X*^+^*DeltaY*^−^*Y*^−^) like their mother (F57). In the balanced Ko12 family sired by the same M5 male when crossed with another female (F25), 28 out of 30 males and 28 out of 30 females had the *amh* (*X*^+^Δ*Y*^−^*Y*^−^) genotype which represents a total of 93.3% individuals carrying the *amh* (*X*^+^Δ*Y*^−^*Y*^−^) genotype characteristic of XX females but half of them actually developed as males. The father’s genotype *amh* (*X*^+^Δ*Y*^−^*Y*^+^) was found in only four individuals (two males and two females).

We also analyzed the genotypic distributions of progenies Ko17, Ko24 and Ko29, all obtained from the female F12 genotyped as *amh* (*X*^+^Δ*Y*^−^*Y*^+^) (Table 7). The full-sib families Ko17 and Ko29 present similar genotypic distributions. The majority of males (97% and 96%) possessed the *amh* (*X*^+^Δ*Y*^+^*Y*^+^) genotype like their father (Table 7; Table S12), which we found generally for XY males. The other males (3 and 4%) had the same genotype as their mother. In the half-sib family Ko24, the majority of males (86%) also had the same *amh* (*X*^+^Δ*Y*^+^*Y*^+^) genotype resembling that of the male breeder. Nevertheless, we encountered 14% males in this family with genotypes like the female F12 mother (Table 7). However, a large proportion of females, 63% for Ko17, 59% for Ko29 and 57% for Ko24, were genotyped as *amh* (*X*^+^Δ*Y*^−^*Y*^+^). Moreover, 10% of the females in the Ko24 had the *amh* (*X*^+^Δ*Y*^+^*Y*^+^) typical of XY males. Nevertheless, a significant proportion of females (32.5% to 33%) were genotyped without both *amh* Δ*Y* and *amhY*, carrying the *amh* (*X*^+^Δ*Y*^−^*Y*^−^) genotype characteristic of XX females.

## Discussion

Genotypic sexing of Nile tilapia has proven to be complicated. The Y chromosome has been located on either LG1 or LG23 depending on the domestic stocks. The male determiner on LG23 was identified as the *amhY* gene, showing high similarities to its X gametolog and to another truncated *amh* gene present on the Y chromosome (Li et al., 2015). In contrast, the causative male gene on LG1 is still unknown (Palaiokostas et al., 2013; Gammerdinger et al., 2014; Palaiokostas et al., 2015; Conte et al., 2017), although two diagnostic SNPs permitted male genotyping in two Manzala strains (Palaiokostas et al., 2013; Wessels et al., 2017).

We have undergone a first study on the sex-determining system in a wild population of Nile tilapia present in Lake Kou located in Burkina Faso. Lake Kou waters belong to the Volta Basin, whose rivers are important reservoirs for Western/Sudano-Sahelian Nile tilapia populations (Trewavas, 1983; Bezault et al., 2011). We focused our study on the LG23 male-determining system because of the existence of diagnostic Y and X chromosome markers based on *amhX*, *amhY* and *amh* Δ*Y* (Li et al., 2015). Despite their highly homologous sequences which complicates the analyses, Li et al. (2015) were able to discriminate XX, XY and YY individuals in the Japanese and local Chinese strains by analyzing different insertions or deletions between the three *amhs*. We tested all the regions used for this discrimination (Sun et al., 2014; Li et al., 2015) but found amongst them only 3 markers which showed clear differences between male and female phenotypes in the Kou fish. We have based our genotype assignments on these 3 *amh* markers and added a fourth marker that detected an *amh* Δ*Y* deletion of 233 bp found in another strain (Eshel et al., 2014). We did not find the diagnostic missense SNP T>C of the *amhY* located in Exon 2 (Li et al., 2015) in the sequences of several wild population (Table S1). This was further confirmed with our KASP analyzes showing that this SNP had only the T allele in both males and female Kou fish.

We genotyped the sex of 91 wild-caught fish from Lake Kou with the four diagnostic *amh* markers. Females had the *amh* (*X*^+^Δ*Y*^−^*Y*^−^) genotype which we identified as XX females, with the exclusion of one female that possessed an *amh* (*X*^+^Δ*Y*^−^*Y*^+^) genotype. The latter together with the other XX genotyped females were all homozygous for the A allele of SNP OniAmh9480 which we have previously found associated to the phenotypic sex in Kpandu fish, another population also from the Volta basin (Table S5). Although this SNP is an intron variant it was strongly correlated with the sexual genotypes assigned in the current study. Several of these females identified as XX were subsequently used as dams in crossings with different genotypic males. When crossed with males assigned as XY males, sex ratios of the offspring were all balanced which validated our genotyping and indicated that they were indeed XX females. However, the sex ratios of the assigned XY female (F12) with the *amh* (*X*^+^Δ*Y*^−^*Y*^+^) genotype were difficult to interpret since they gave both balanced and male-skewed sex ratios depending on who this dam mated with. In this last crossing we did not manage to identify YY individuals using the *amh* genotyping. Likewise, the male (M5) with the same *amh* (*X*^+^Δ*Y*^−^*Y*^+^) genotype based on his sired offspring behaves either like an XY or an XX male. This *amh*-genotype suggests that *amh* Δ*Y* and *amhY* are not always in tandem in the Kou individuals contrary to what has been previously observed in the Japanese strain (Li et al., 2015). Our results may be due to recombination occurring between the X and Y chromosome in these individuals. An alternative, is that *amhY* might act in conjunction with the *amh* Δ*Y* gene to determine sex.

It is still not clear how the two *amh* gametologs (*amhX* and *amhY*) together with the *amh* Δ*Y* function. It is possible that both *amhY* and *amh* Δ*Y* ensure high *amh* expression in the gonad at a critical stage. Because of a shift in the reading frame of *amh* Δ*Y* with a premature stop codon it is thought to give a truncated *amh* protein lacking the transforming growth factor β (*TGF-*β) domain (Eshel et al., 2014; Li et al., 2015). The *TGF-*β domain is known to be necessary for binding *amhs* to their receptor *amhrII* (Li et al., 2015; Zheng et al., 2018). Therefore, only *amhY* might be the ligand of *amhrII* (Li et al., 2015). Knockout of *amhY* or both *amhY* and *amh*Δ*Y* caused male-to-female sex reversal, while mutation of *amh*Δ*Y* alone apparently could not (Li et al., 2015). Hence, *amhY* alone could be a functional Y-linked marker and under that hypothesis, the individuals displaying *amh* (*X*^+^Δ*Y*^−^*Y*^+^) genotype would be XY. Hence, the Kou female would be a spontaneously sex-reversed XY female. Nevertheless, some studies highlighted the *amh*Δ*Y* gene as a putative Y gene in a Manzala strain (Eshel et al., 2014; Wessels et al., 2017). In this last case, both male and female showing *amh* (*X*^+^Δ*Y*^−^*Y*^+^) genotypes would be XX individuals and the male could be naturally sex-reversed. In addition, the identification of the two *amh* gametologs (*amhX* and *amhY*) together with the *amh* Δ*Y* has been based on the absence and/or presence of some deletions an/or insertions, and this may not be sufficient to conclude for the absence of a gene and subsequently the absence of a sex chromosome, because recombination patterns between the X and the Y chromosome could be involved (Wessels et al., 2017). Alternatively, polymorphisms may exist in the *amhY* or the *amh*Δ*Y* sequences.

On the other hand, we found a high proportion of females genotyped as *amh* (*X*^+^Δ*Y*^−^*Y*^+^) among the G1 progenies obtained from the female F12 carrying the *amh* (*X*^+^Δ*Y*^−^*Y*^+^) genotype. It is possible that sex in progenies obtained from F12 and the male M5 was driven by the dosage levels regarding a sex threshold. Perhaps the *amhY* alone without *amh* Δ*Y* did not reach the threshold necessary to induce masculinization. Alternatively, dosage levels might be brought by other genes from the pathway or network of sex determination or differentiation. As proposed by Perrin (2016), it is conceivable that under some rare conditions (e.g., specific genotypes or environments) any fluctuations or dosage in the expression of key genes could exceed a sex-threshold and drive the development towards the unexpected phenotypic sex. Additional research is required to shed light on the existence of such a threshold-effect as well as the existence of possible recombination amongst the *amhs*.

The majority of the Kou males (67.4%) had the *amh* (*X*^+^Δ*Y*^+^*Y*^+^) genotype and were considered as XY males. However, a very high proportion (30.4%) of males were assigned as XX males due to their *amh* (*X*^+^Δ*Y*^−^*Y*^−^) genotypes. These genotyped XX males were, like the females, AA homozygotes for the OniAmh9480 whereas males genotyped as XY were AC heterozygotes. These genotyped XY fish sired normal expected balanced sex ratios. In contrast, most of the males genotyped as XX did not sire offspring with the expected all-female or female-skewed sex ratios, but instead gave balanced sex ratios. Our results indicate that the *amh*-genotype assignment was not sufficient for these XX-males and that sex is controlled by another locus/other loci which may be epistatically dominant over the LG23 locus. Because previous results have shown LG1 to be associated to sex in certain strains (Cnaani et al., 2008; Palaiokostas et al., 2013; Gammerdinger et al., 2014), we should look for this locus or loci notably on LG1. In contrast, we were able to demonstrate with the X and Y markers from LG23 the finding of one XX male. We have therefore shown the existence of at least one naturally female-to-male sex reversed fish in the Kou population.

Sex reversal induces a mismatch between the genotypic and phenotypic sex (Baroiller & D’Cotta, 2016). Sex in the Nile tilapia has been shown to be governed not only by a pair of sex chromosomes but also by parental and environmental factors (Baroiller et al., 1995; Baroiller & D’Cotta, 2001; Wessels & Hörstgen-Schwark, 2007; Cnaani et al., 2008). While genetic factors considered as minor brought by both parents might have had effects on the sex in the Kou fish for instance in the XY putative female, Kou XX males could result from high temperatures. Temperatures >32 °C acting on sex-differentiating stages have been shown to induce female to male sex reversal in the Nile tilapia (Baroiller et al., 1995; Baroiller et al., 2009; Baroiller & D’Cotta, 2016). Individuals from Lake Kou have indeed been shown to be sensitive to temperature sex-reversal (Table S13). Thermosensitivity in Nile tilapia is a heritable trait brought by both the father and mother genomes and can be selected in only three generations to produce a highly thermosensitive line (∼93% male proportions) in the Manzala-Göttingen strain (Wessels & Hörstgen-Schwark, 2007; Wessels & Hörstgen-Schwark, 2011). Mapping studies revealed that LG1, LG3 and LG23 were associated to the XX males in this line (Lühmann et al., 2012; Wessels et al., 2014). RAD analyses has confirmed the strong association of LG23 (Wessels et al., 2017) whereas, XX males of the Manzala-Stirling strain showed strong association to LG20 (Palaiokostas et al., 2013; Palaiokostas et al., 2015). Our results further indicate that within the Manzala strain, sex determination has evolved differently in the various laboratories to which the strain was transferred. Whereas the Manzala fish at the University of Stirling have the LG1-XY system (Palaiokostas et al., 2013), ours from Tihange (Belgium) have the LG23-XY system and this is also the case for the Manzala-Göttingen stock (Germany) (Wessels et al., 2017), although these last two derived 33 years ago from the Stirling stock (D Penman & C Mélard, pers. comm., 2017). The Manzala strain was bought by Tihange in 1986 and it potentially represents about thirty successive generations since their arrival (C Mélard, pers. comm., 2017).

We cannot exclude the effects of xenobiotics from industrial waste or agricultural pesticides causing sex-reversals (Brown et al., 2015) in Lake Kou. However, both temperature sex reversals and parental effects have been identified in several experimental conditions where the 27 °C controls showed no sex-reversals. Moreover, sex reversals have also been suggested in wild populations living in Ethiopian Lakes where no urban or agricultural wastes occur. In this last study, microsatellite markers particularly for LG1 suggested but could not confirm the presence of naturally sex reversed XX males in significant proportions and also XY females to a lesser extent in the wild populations of Koka found in the Awash basin (Ethiopia) and of Kpandu from the Volta Bassin (Ghana). Progeny testings further suggested that these fish were wild sex-reversed individuals (Bezault et al., 2007).

Our study indicates that sex-determination in wild Kou fish appears to be more complicated than what is seen in some domestic stocks with the probable existence of several sex-determining loci interacting. We hypothesize that genetic bottlenecks or drift, inbreeding, selection and/or hybridization of domesticated strains might have increased the frequency of lethal or semi-lethal alleles resulting in reduced variability of some genotypes. It is also possible that these (fortuitous or not) genetic events have reduced or eliminated polymorphisms at some sex-determining loci, so that only the XY locus located on either LG23 (i.e., in the Japanese strain) or LG1 (i.e., in the Manzala-Stirling strain) have been retained. Alternatively, these loci might still exist but they are repressed or weaker than LG1 or LG23 Y-loci. Thus, Kou individuals displaying unpredictable genotypes in relation to their phenotypes could be associated to another/other linkage group(s) and consequently more markers than just those of the *amhs* on LG23 are required. Genotyping of two SNPs located on LG1 (Oni23063 and Oni 28137) that were found to be highly correlated to the phenotypic sex in Manzala fish (Palaiokostas et al., 2013; Wessels et al., 2017) failed to be associated to Kou males and females. We have found in Lake Kou fish that sex is nevertheless strongly correlated to the *amh* sex-linked markers for a significant proportion of analyzed individuals, suggesting that *amhY* is at least one of the key genes for sex determination in this population. Genomic approaches might shed light on what other LGs are interacting.

## Conclusions

We have genotyped the sex in a wild population of Nile tilapia for the first time using the sex-determining *amhY* gene, its *amhX* gametolog together with *amh* Δ*Y*. Correct assignments of the genotype with the male and female phenotypes was observed for 82% of the individuals, suggesting that the *amhY* locus on LG23 is either the main male-determiner or at least a key gene for sex determination in the Kou population. Mismatch between the genotype and the sexual phenotype in the rest of the fish strongly suggest that another locus or other loci is/are also governing sex. We have found at least one XX male in the Kou Lake. It is necessary to enlarge the study of wild populations of Nile tilapia to see whether sex is strongly determined by the *amhY*-LG23 gene and define the frequencies of sex-reversal encountered in these wild populations.

##  Supplemental Information

10.7717/peerj.7709/supp-1 Figure S1 Electrophoretic gels obtained from four amh markers for wild fish from Lake Kou and for two domestic strains (Manzala and Japanese) of Nile tilapia Raw data. The 1,000 bp amplification with *amhX*_+36_ is X chromosome specific. *AmhY*_−233_ is associated with a 1,000 bp for X and Y amplification and a ∼800 bp for the Y chromosome. *AmhY*_+5_ is Y specific and *amhY*_−5608_ is X (8,022 bp) and Y (2,414 bp) chromosome specific. Manz. = Manzala, Jap. = Japanese and Ld = DNA ladder.

10.7717/peerj.7709/supp-2 Figure S2 Electrophoretic gels obtained from amh markers for G1 individuals Raw data.The 1,000 bp amplification with *amhX*_+36_ is X chromosome specific. *Amh*Δ*Y*_−233_ is associated with a 1,000 bp for X and Y amplification and a 800 bp for the Y chromosome. *AmhY*_−5608_ is X (8022 bp) and Y (2,414 bp) chromosome specific. Ld = DNA ladder.

10.7717/peerj.7709/supp-3 Table S1 Sanger sequences showing the SNPs identified between sexes or populations The SNP is named according to the sequence TTATATTTATG considered.

10.7717/peerj.7709/supp-4 Table S2 Water quality in Lake Kou, well water and the experimental facilties during the key stages of the fish life cycle Data are represented as Mean value ±Standard deviation.

10.7717/peerj.7709/supp-5 Table S3 Temperature, pH and dissolved oxygen survey in Lake Kou for a 24-day period recorder 4 times per day, in 3 differents areas at 5 and 20 cm depth Raw data. Figure of pH values recorder showing two extreme values that were not in the common range and therefore we have eliminated them.

10.7717/peerj.7709/supp-6 Table S4 Temperature, pH and dissolved oxygen survey during the different stages of the progeny testing performed under controlled experimentations Raw data.

10.7717/peerj.7709/supp-7 Table S5 Association of the SNP OniAmh9480 to sex in Lake Kpandu population Lake Kpandu is located in Ghana and shares the same Volta basin as Lake Kou, draining into the Volta River.

10.7717/peerj.7709/supp-8 Table S6 SNP markers used for KASP assay Primers’ sequences, localisation of the markers, and the references are indicated.

10.7717/peerj.7709/supp-9 Table S7 Survival and mortality rates of progenies tested showing the sex ratios observed in the survivals at 90 dpf Sampling details and the mortality due to technical failures are shown.

10.7717/peerj.7709/supp-10 Table S8 Frequency of stocking density, mortality, number of fish sexed and the male proportions of the progenies tested Stocking density = Number per aquaria/water volume of aquaria. Mortality frequency = Mortality number/Initial Number. Frequency of fish sexed= Number of sexed fish/Survival number. Males frequency = Number of males.

10.7717/peerj.7709/supp-11 Table S9 Amh-genotype analysis with the corresponding genotype assignment and the alleles values obtained with the KASP assay for OniAmh9480 Raw data for 91 Kou breeders.

10.7717/peerj.7709/supp-12 Table S10 Sex-ratio stabillity independently of the mortality rate The repeated crossings with the same Kou parents demonstrate that mortalities are not sex-biased.

10.7717/peerj.7709/supp-13 Table S11 Evidence that induced mortalities (simulated by hypoxia treatment) after sex differentiation are not sex specific Raw data. The dead fish due to technical problem (hypoxia) were sexed immediately upon death. Then 50 fish were sampled among survivors, sacrificed and sexed. After sampling, the survivors were also sacrificed and sexed. Sex-ratio are compared using Fischer exact test. The detailed table of sexing, the summary table of sexing, the statistical analysis and the variation of dissolved oxygen in the aquaria during the technical problem, are shown.

10.7717/peerj.7709/supp-14 Table S12 Amh-marker analyses of KOU G1 individuals Raw data. The presence or the absence of *amhX, amhΔY* and *amhY* are indicated.

10.7717/peerj.7709/supp-15 Table S13 Effect of water temperature during sex differentiation (∼10 to 40 dpf) on the sex-ratio of Lake Kou Nile tilapia Three males with nine females were stocked in a 3 m^3^ concrete basin and 30 days after, we collected eggs and larva. According to the development stages described by FujimuraOkada2007, we selected the larva which were at the stages 25 to 27, and transferred them to 2 ×300 L aquaria where they were treated at 27 ±2° C 36 ±2° C respectively during 30 days. After the thermal treatment, they were reared at 27 ±2° C for 60 days. 320 fish per treatment were sacrificed for sex-ratio analysis at ∼90 dpf.

## Additional Information and Declarations

### Data Availability

The following information was supplied regarding data availability: The raw data are available in the Supplemental Files.
