# Supplementary figures and images for: Mismatches between the genetic and phenotypic sex in the wild Kou population of Nile tilapia Oreochromis niloticus
Source: PeerJ. 2019 Sep 18;7:e7709. doi: 10.7717/peerj.7709 (PMC6754722; doi:10.7717/peerj.7709)

amhX<sub>+36</sub>

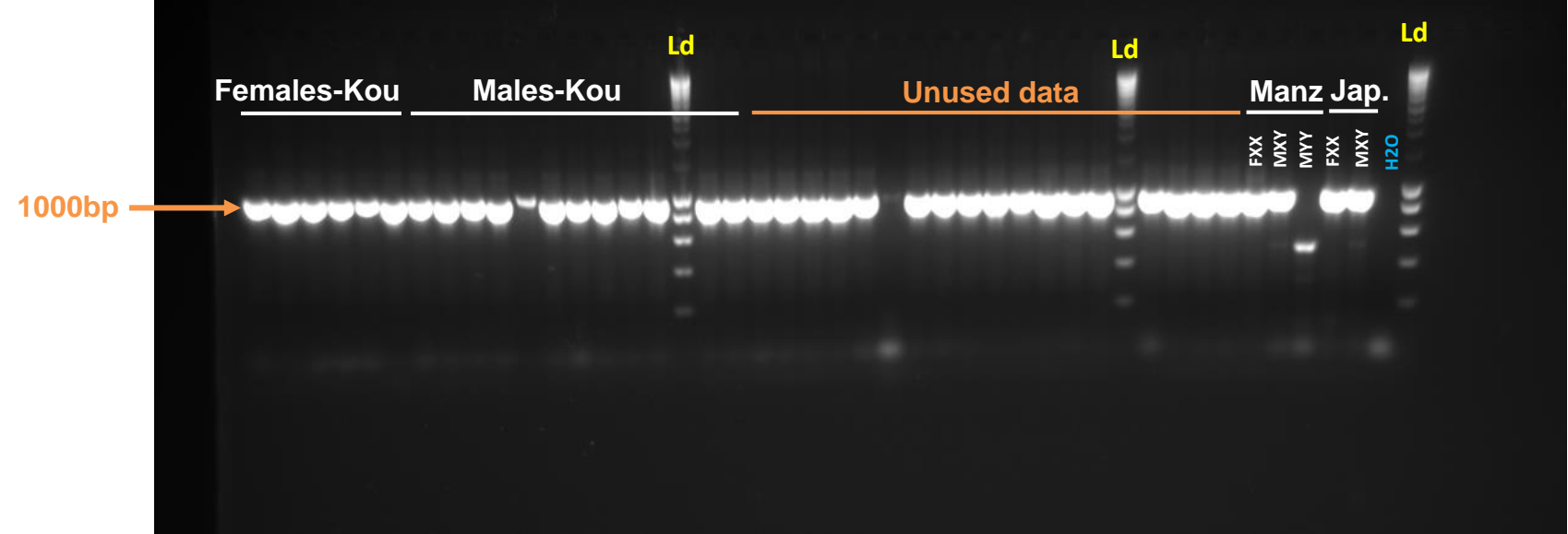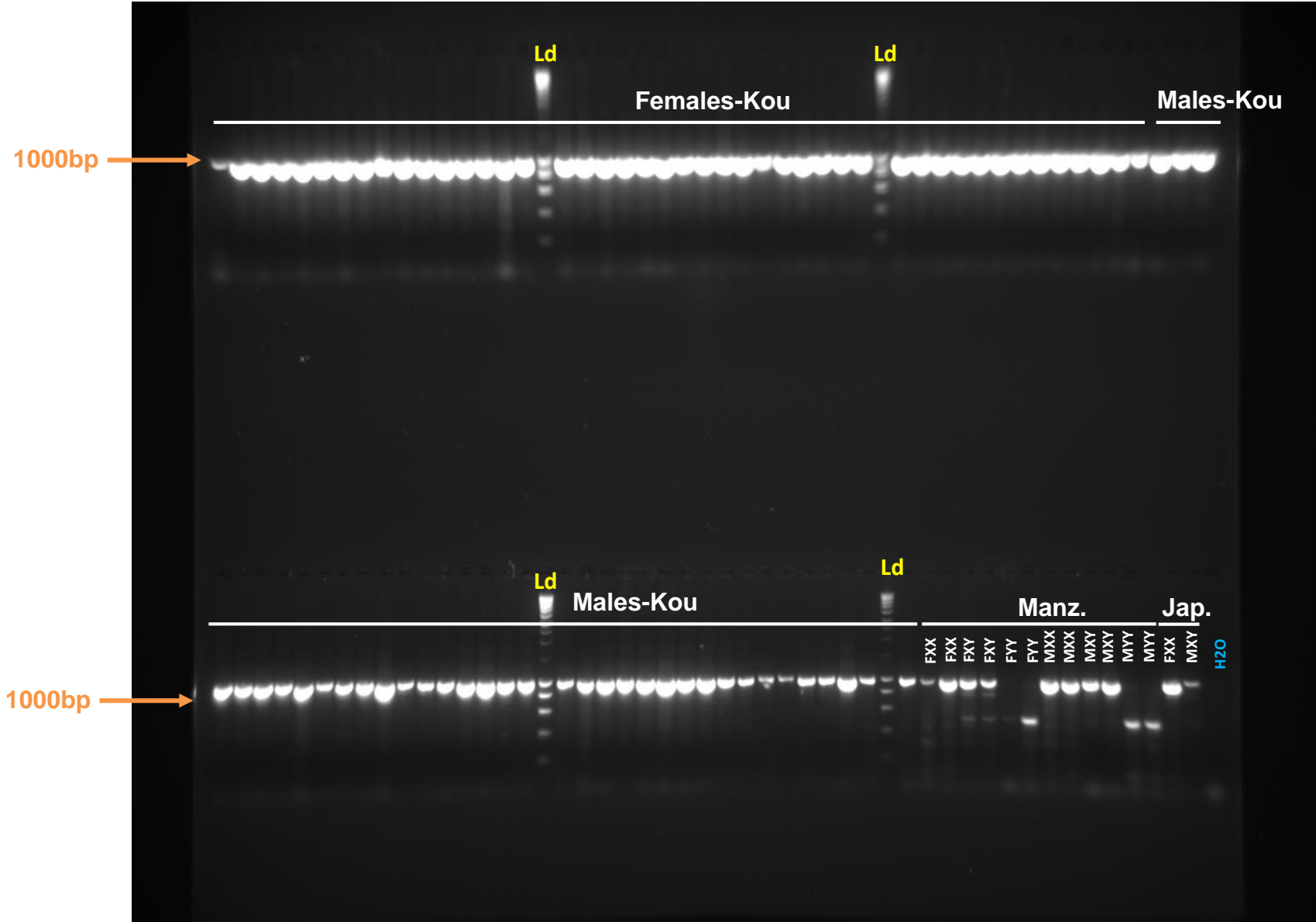

amhΔY<sub>-233</sub>

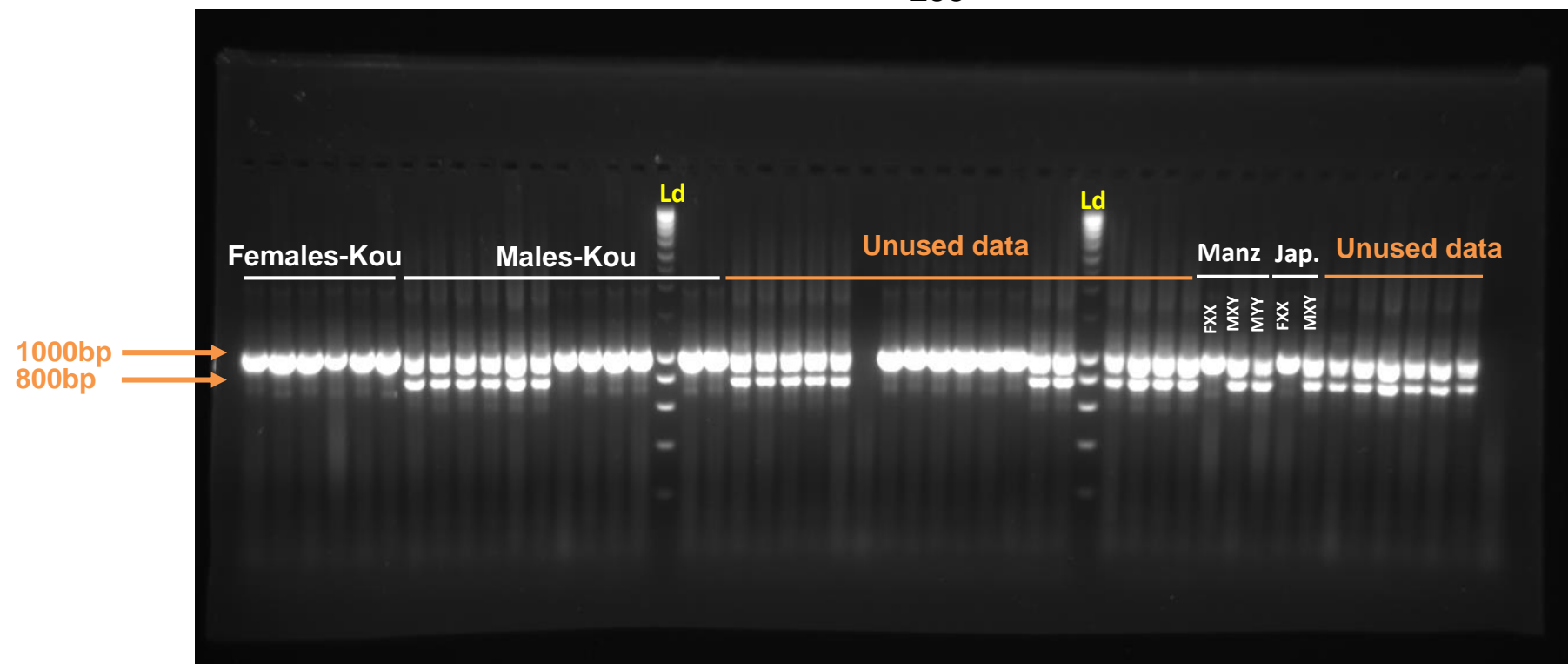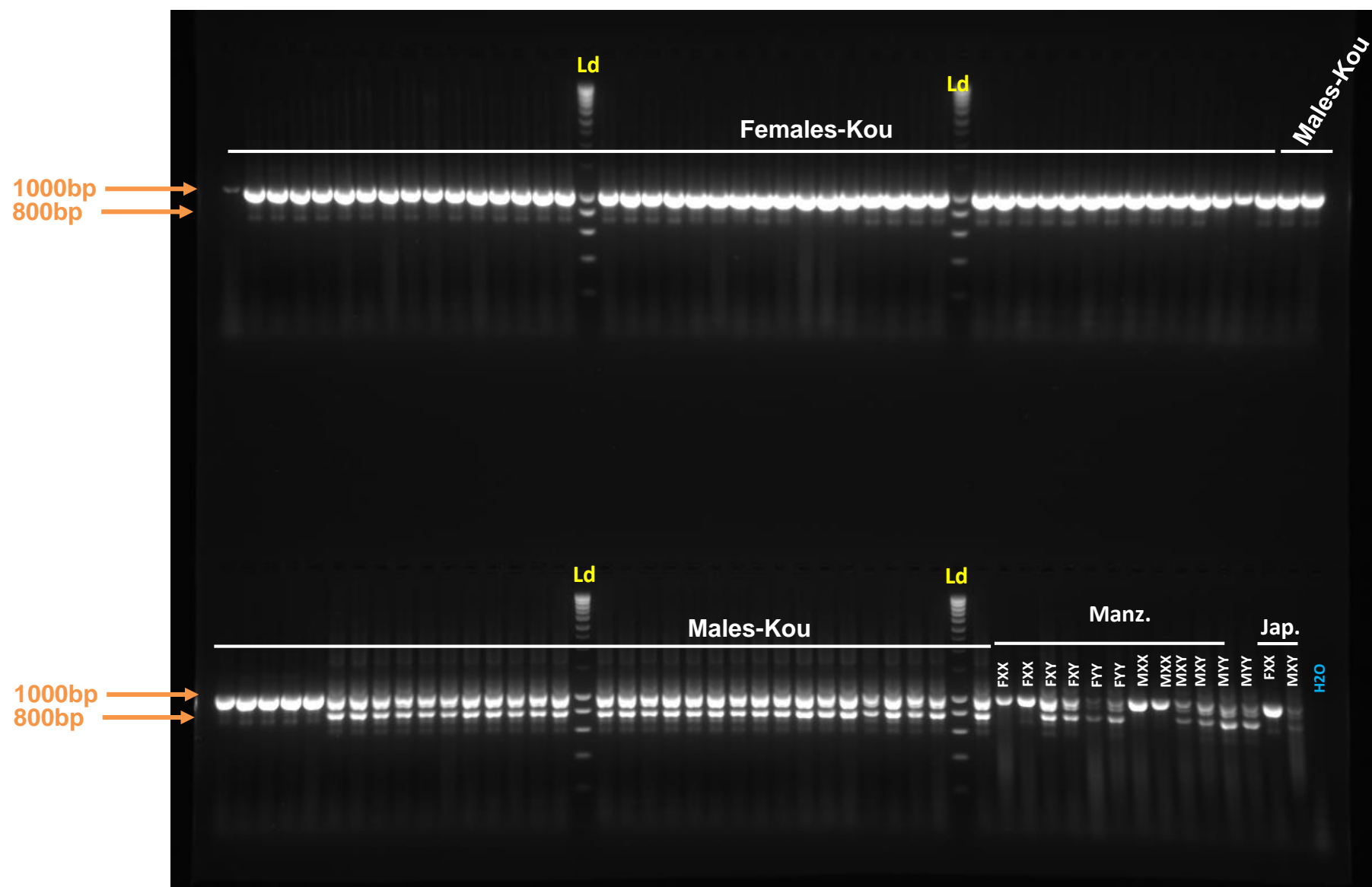

amhΔY<sub>+5</sub>

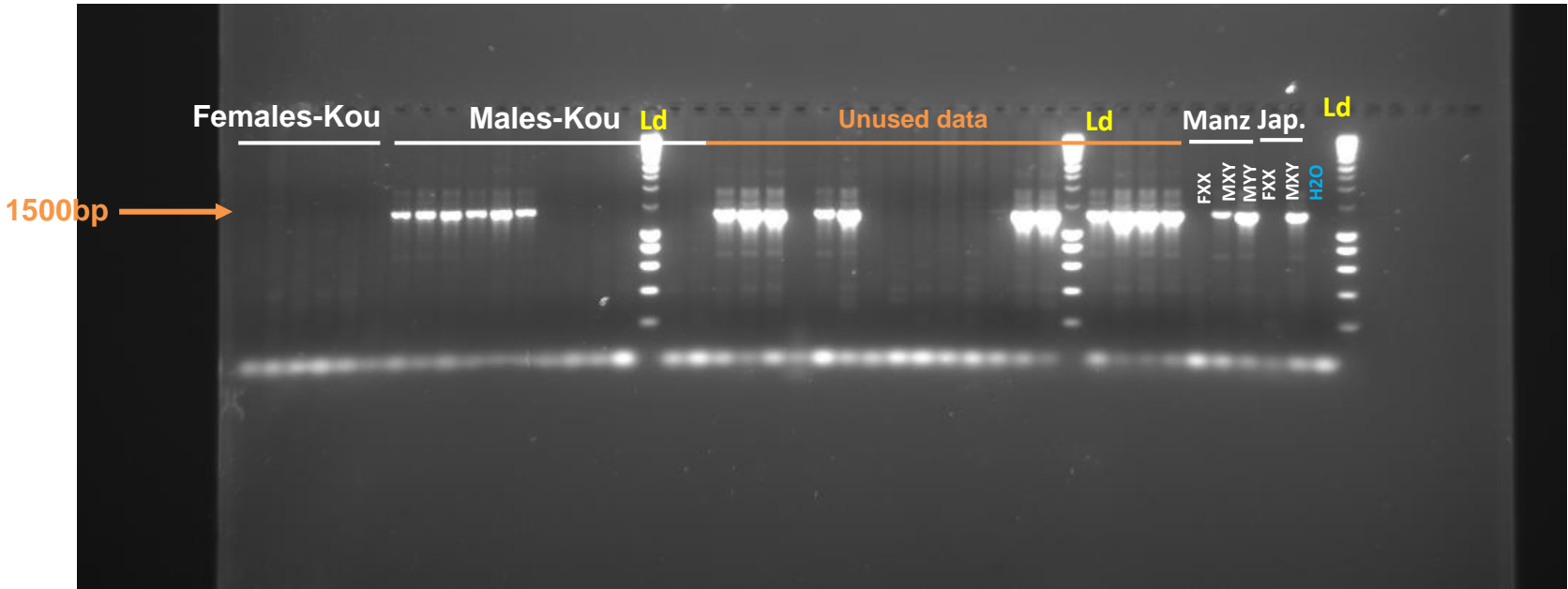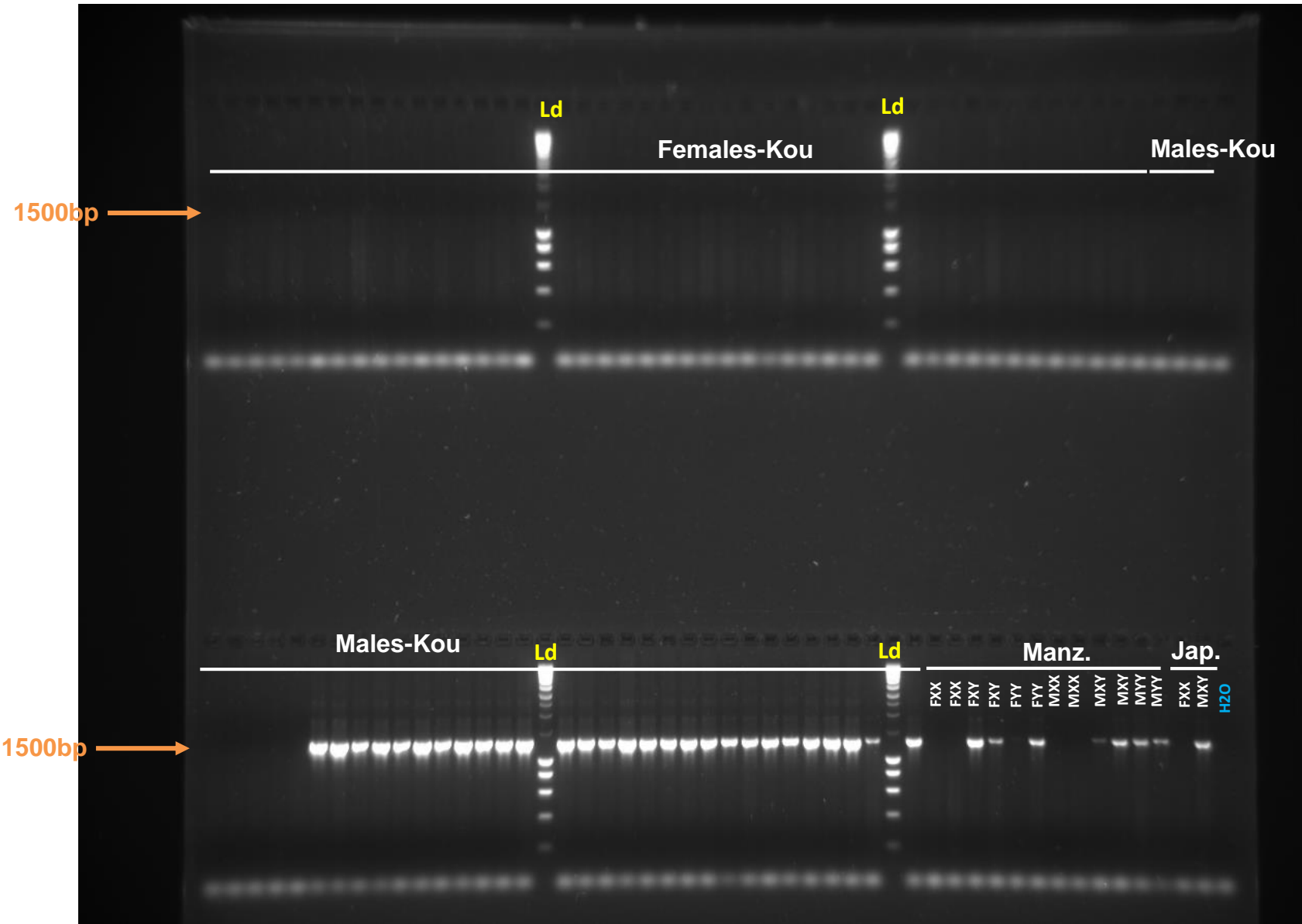

amhY<sub>-5608</sub>

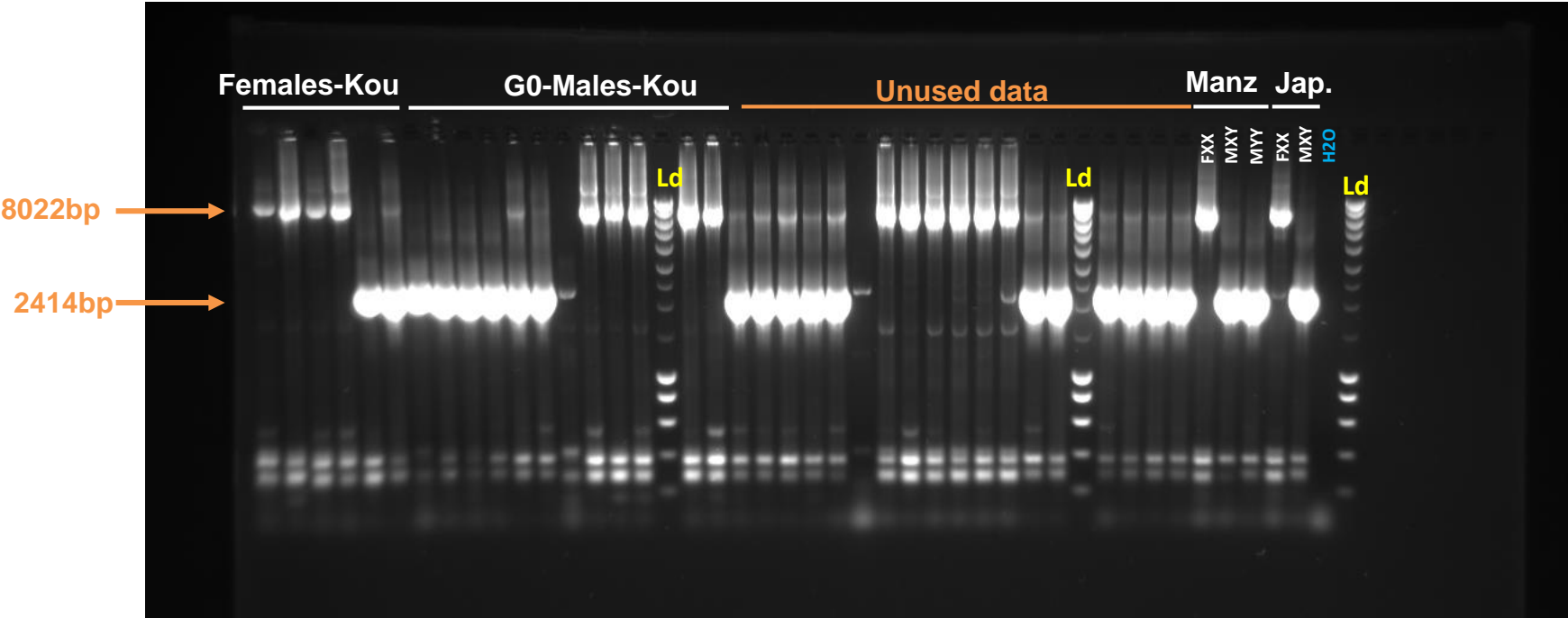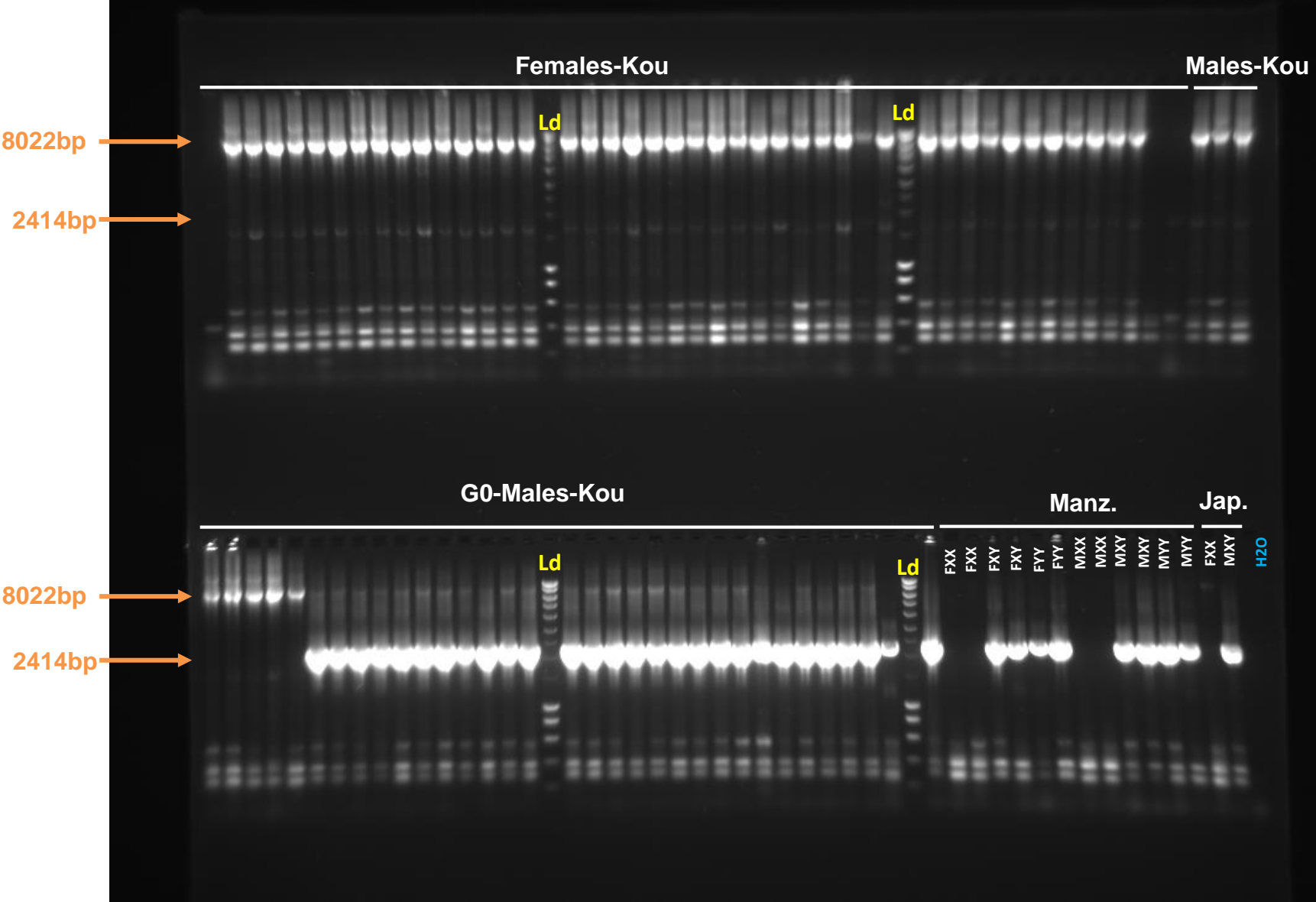

Supplement: Figure S1 — Raw data. The 1,000 bp amplification with amhX+36 is X chromosome specific. AmhY−233 is associated with a 1,000 bp for X and Y amplification and a ∼800 bp for the Y chromosome. AmhY+5 is Y specific and amhY−5608 is X (8,022 bp) and Y (2,414 bp) chromosome specific. Manz. = Manzala, Jap. = Japanese and Ld = DNA ladder. [file peerj-07-7709-s001.pdf]
