# Supplementary material for: Mismatches between the genetic and phenotypic sex in the wild Kou population of Nile tilapia Oreochromis niloticus
Source: PeerJ. 2019 Sep 18;7:e7709. doi: 10.7717/peerj.7709 (PMC6754722; doi:10.7717/peerj.7709)
Supplement: Figure S2 — Raw data.The 1,000 bp amplification with amhX+36 is X chromosome specific. AmhΔY−233 is associated with a 1,000 bp for X and Y amplification and a 800 bp for the Y chromosome. AmhY−5608 is X (8022 bp) and Y (2,414 bp) chromosome specific. Ld = DNA ladder. [file peerj-07-7709-s002.pdf]

Family Ko19 females

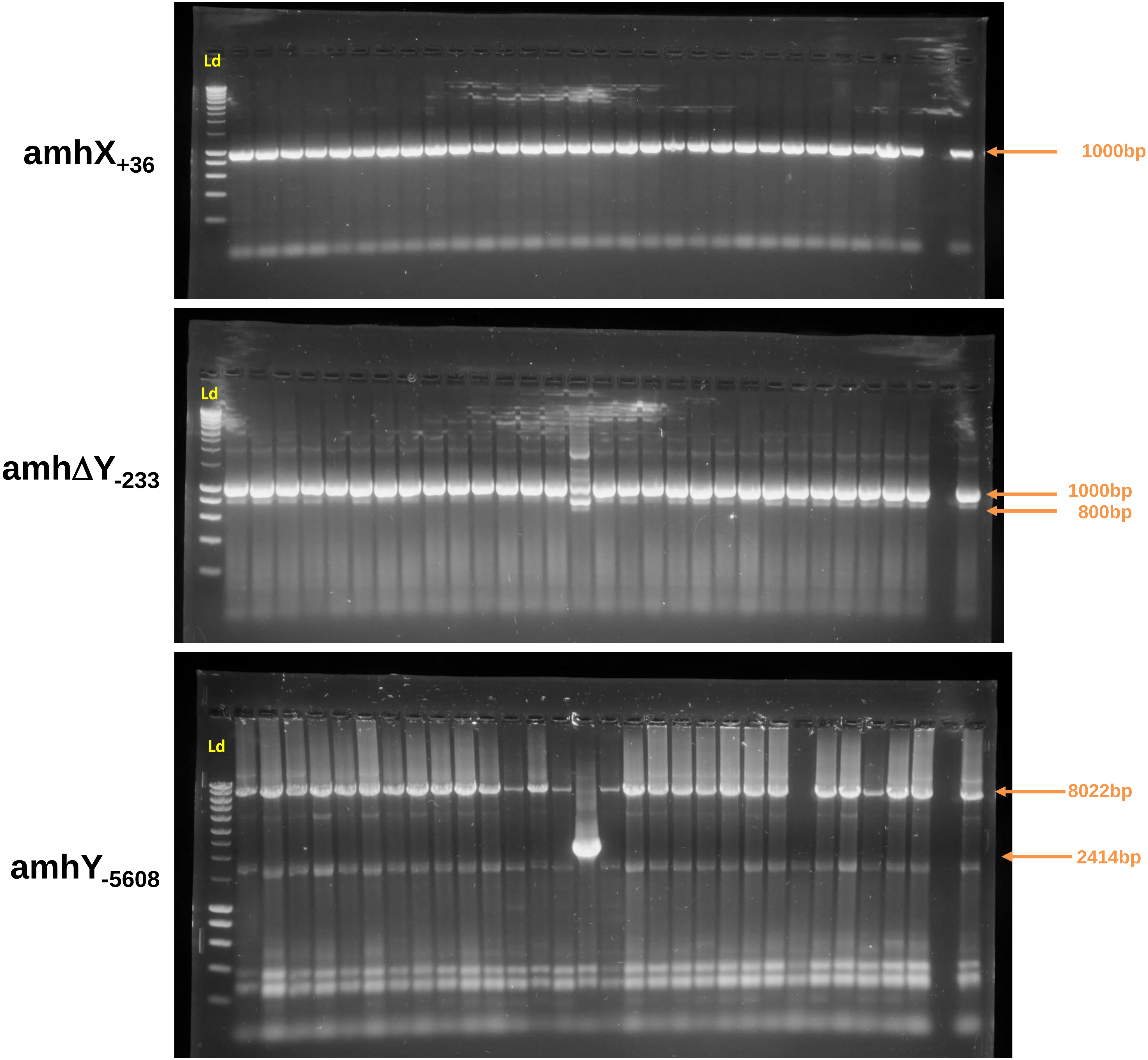

Family Ko19 males

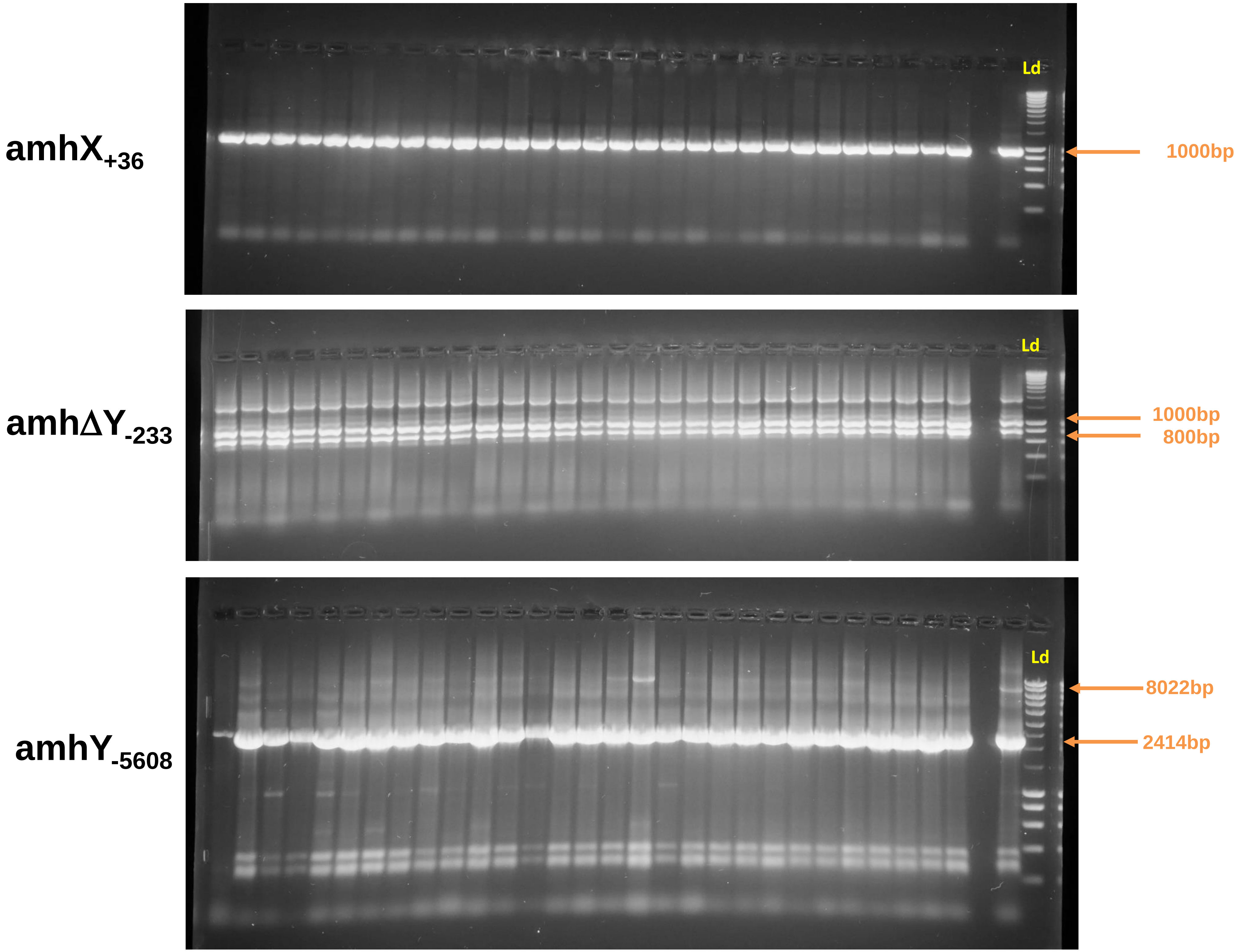

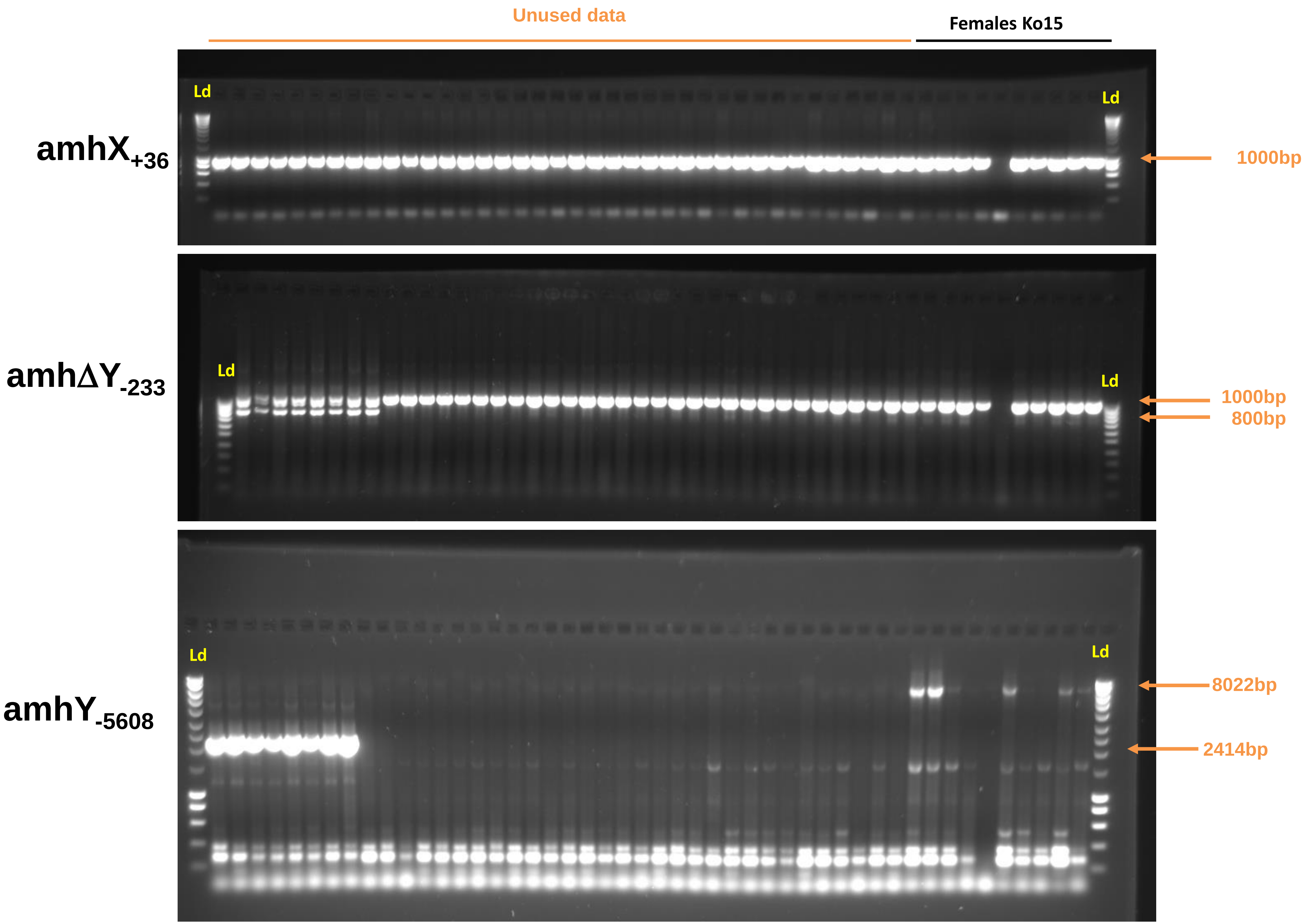
Unused data
Females Ko15
amhX+36
Ld
Ld
1000bp
amhΔY-233
Ld
Ld
1000bp
800bp
amhY-5608
Ld
Ld
8022bp
2414bp

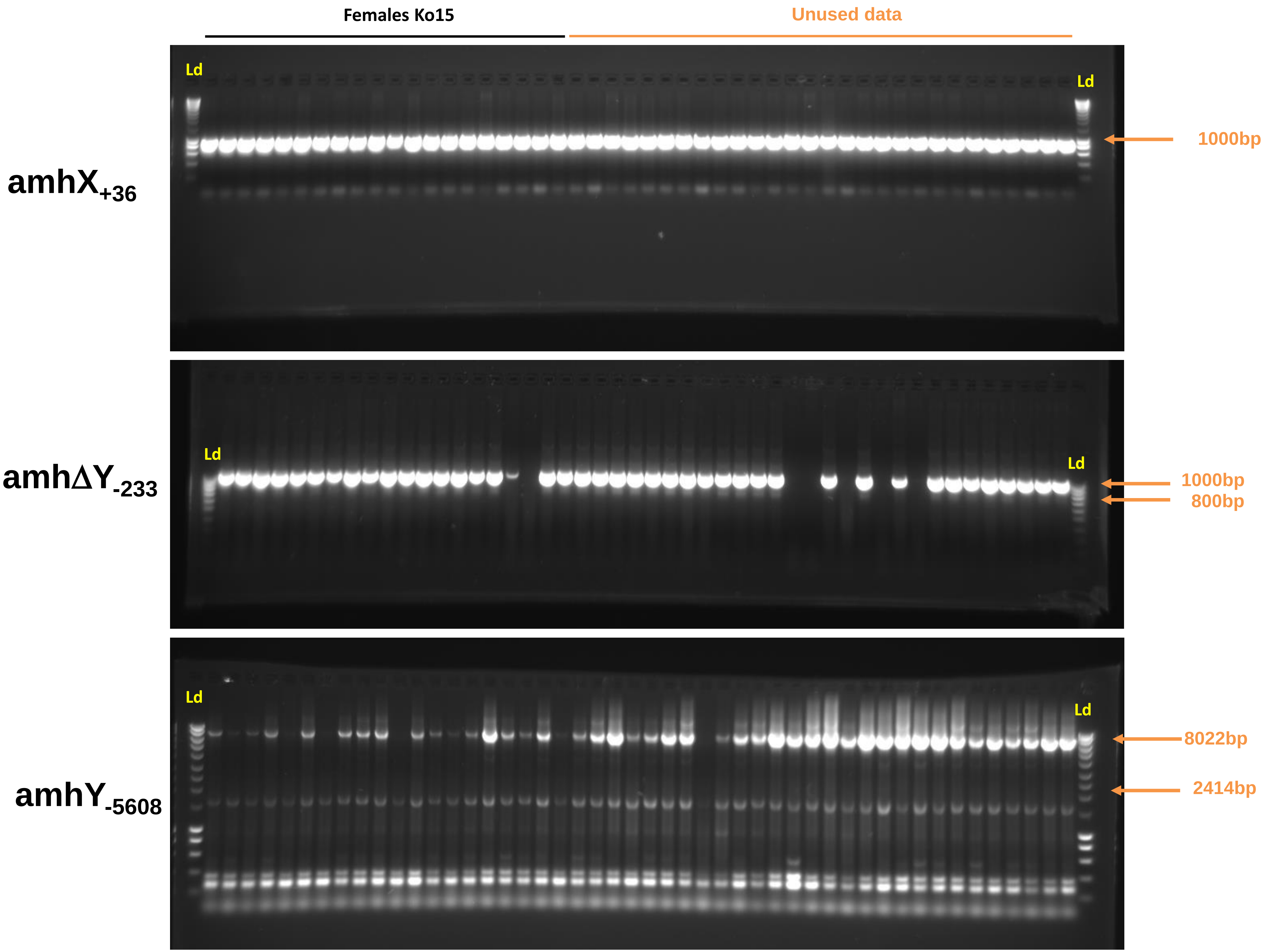

Females Ko15
Unused data
Ld
Ld
amhX+36
1000bp
Ld
Ld
amhΔY-233
1000bp
800bp
Ld
Ld
amhY-5608
8022bp
2414bp

Family Ko22 females

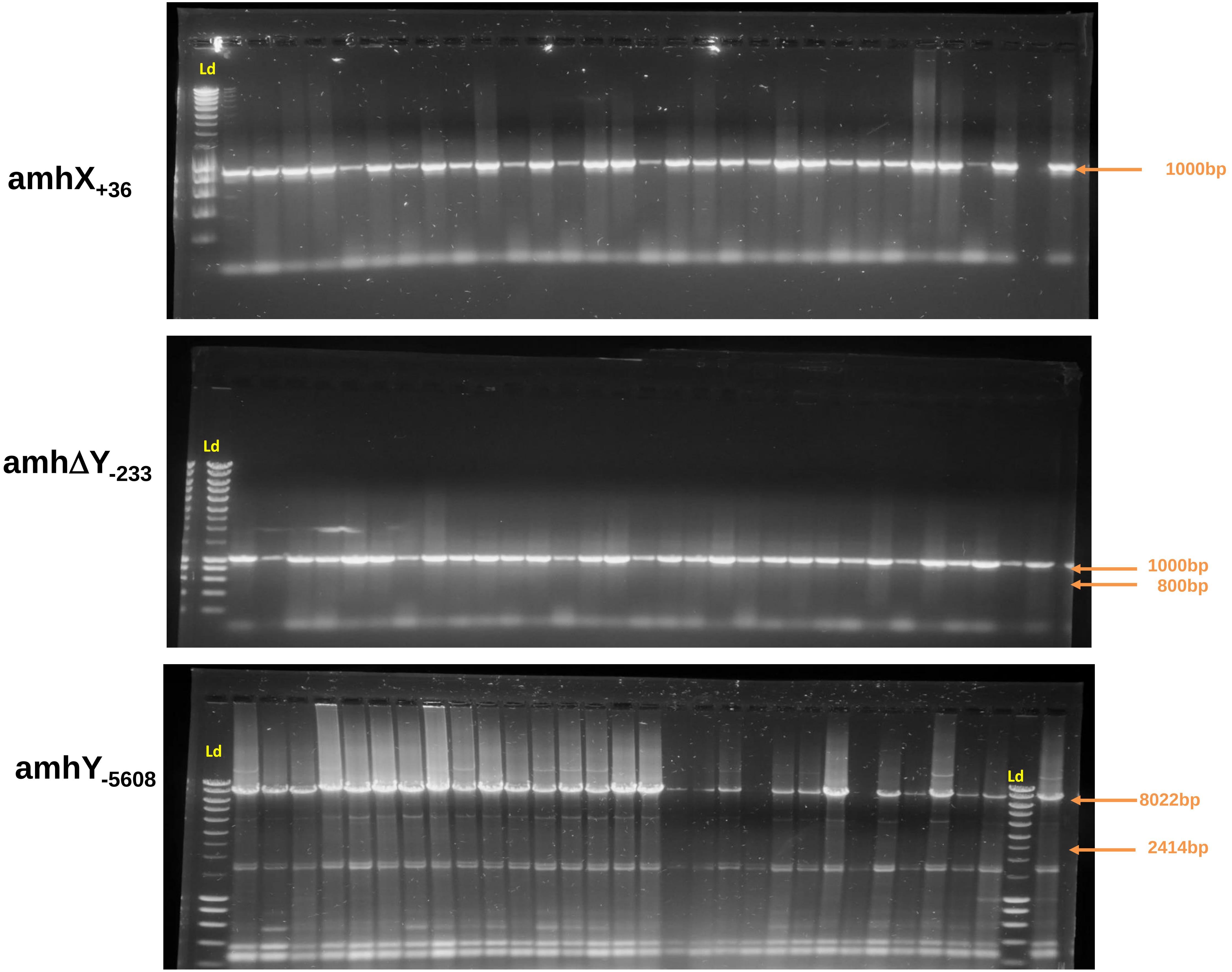

Family Ko22 males

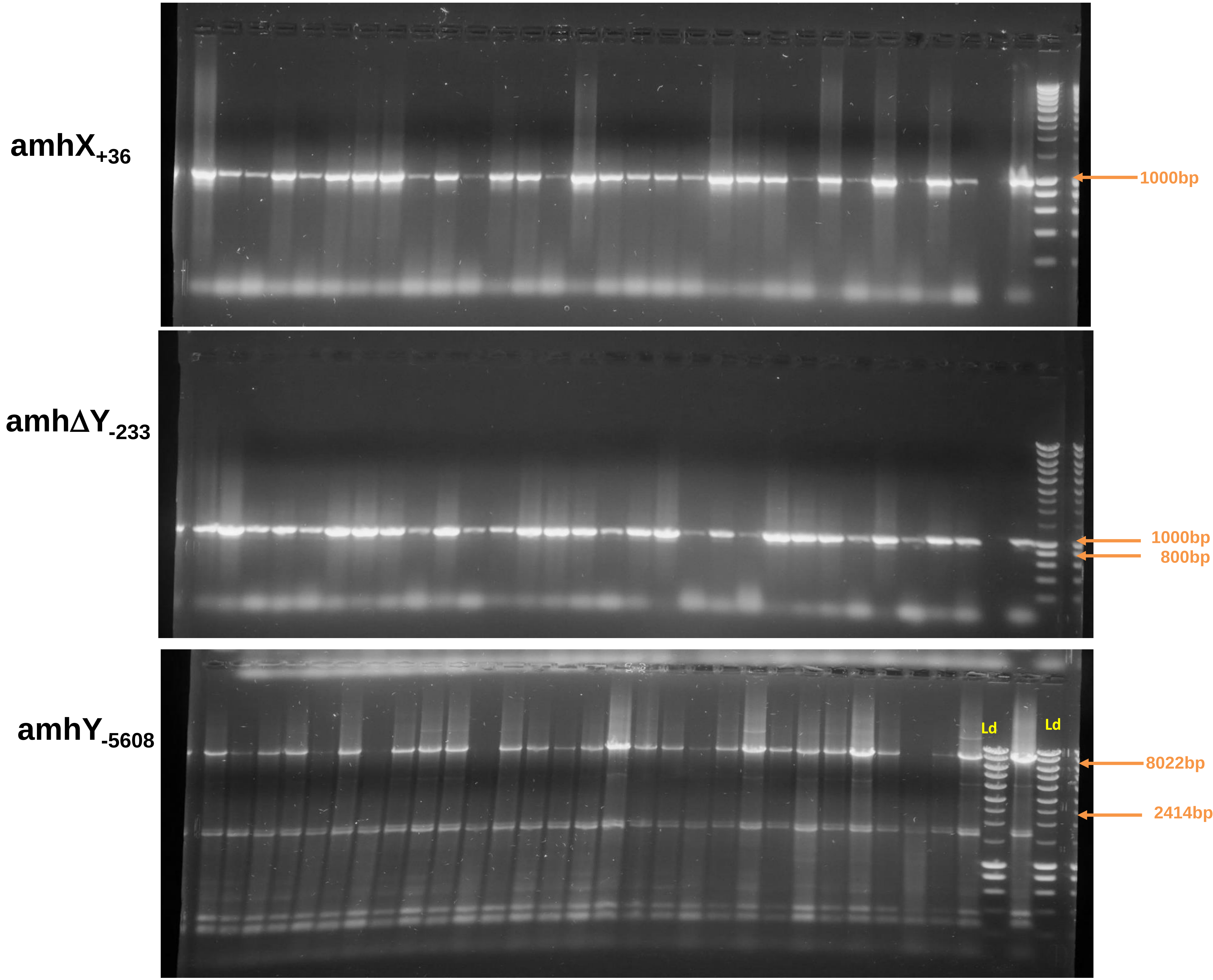

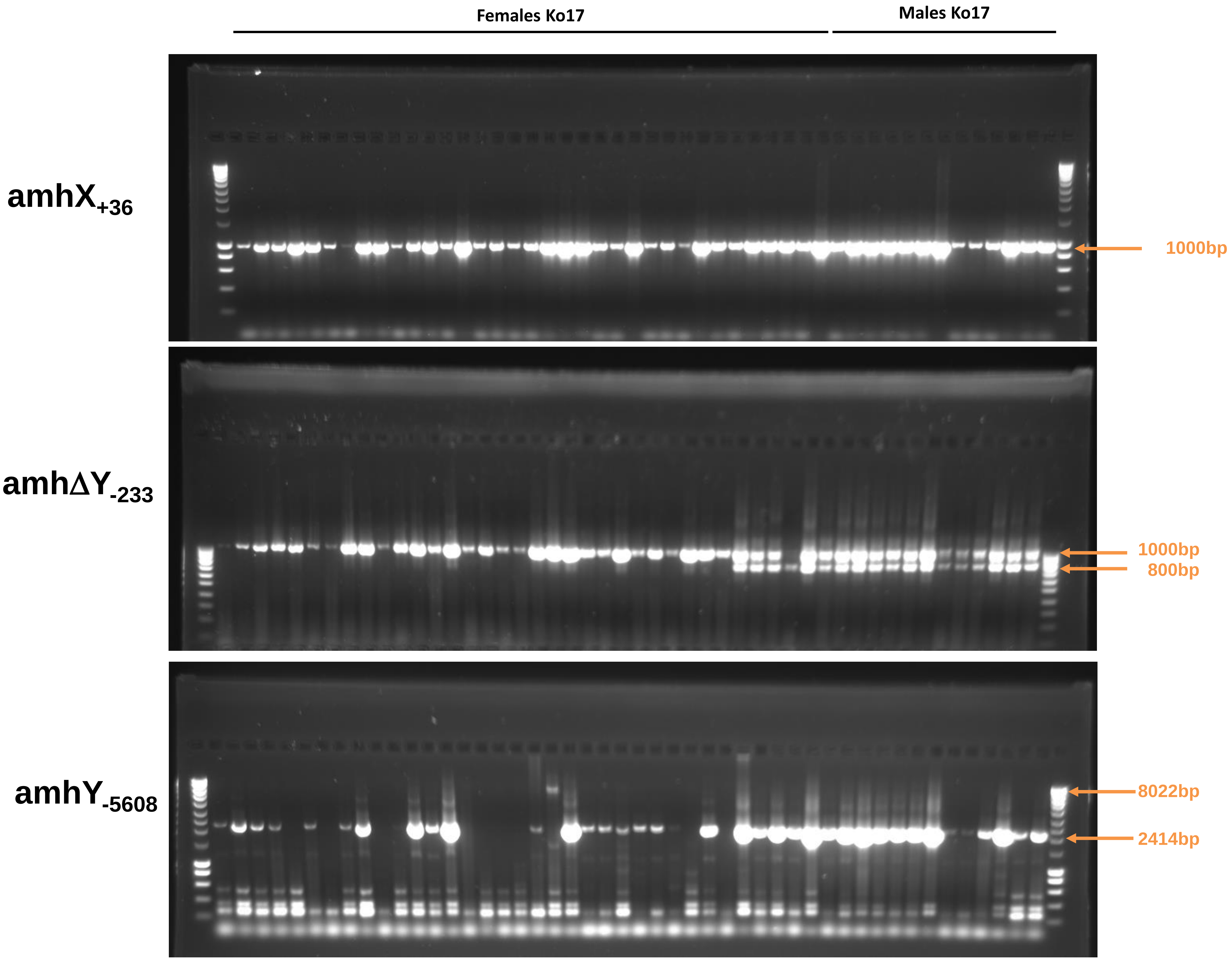

Females Ko17
Males Ko17
amhX+36
1000bp
amhΔY-233
1000bp
800bp
amhY-5608
8022bp
2414bp

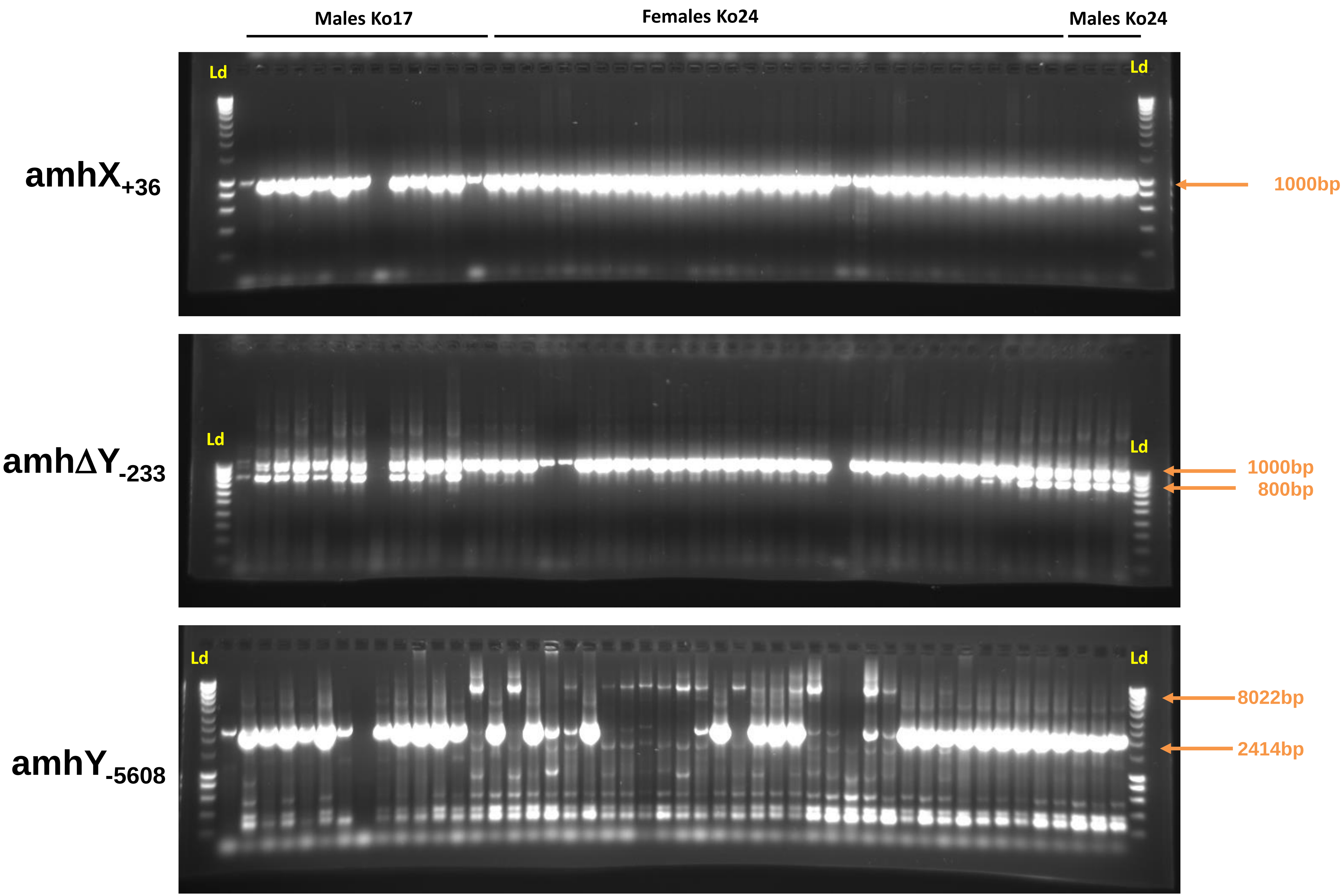
Males Ko17
Females Ko24
Males Ko24
amhX+36
Ld
Ld
1000bp
amhΔY-233
Ld
Ld
1000bp
800bp
amhY-5608
Ld
Ld
8022bp
2414bp

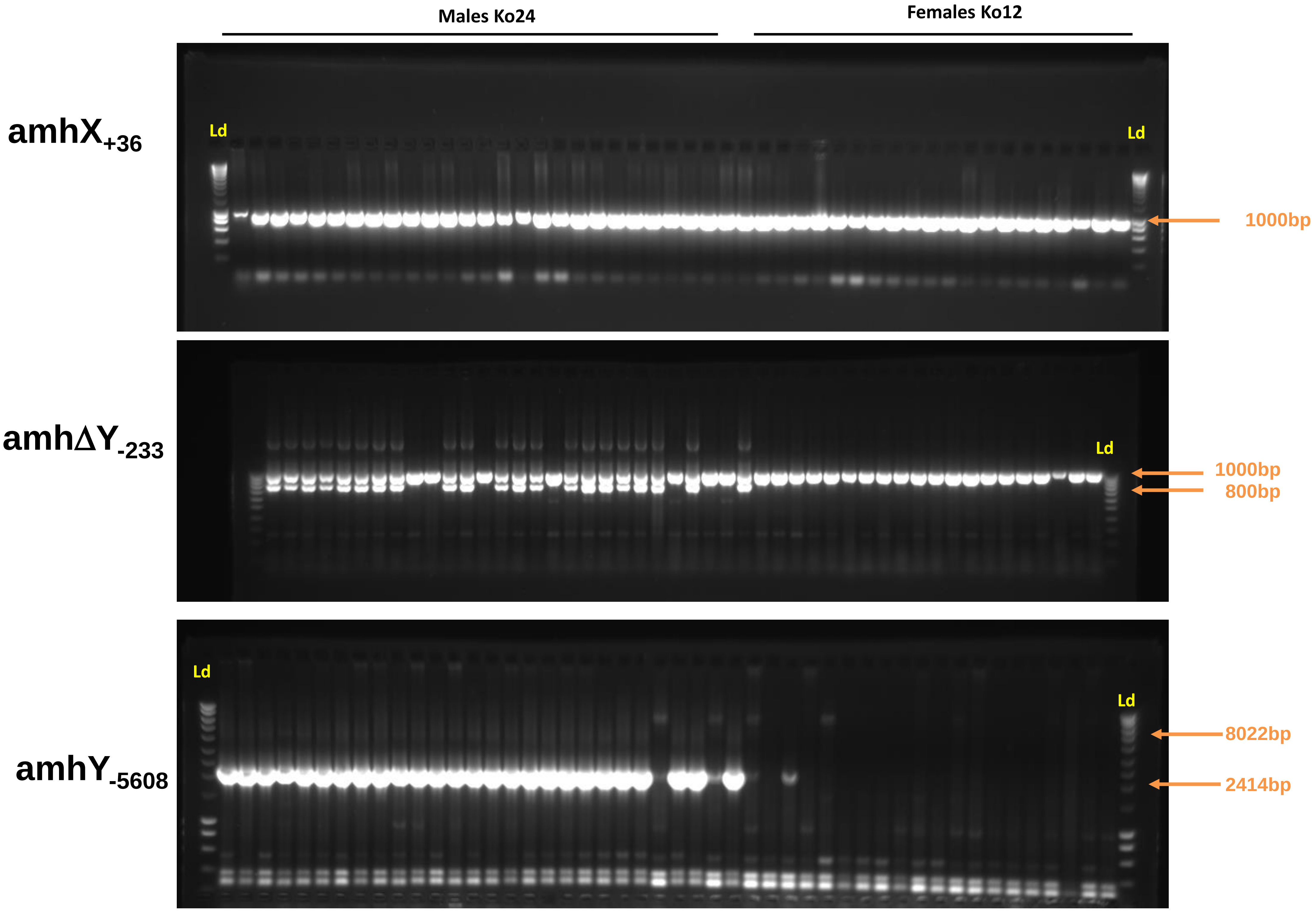

Males Ko24
Females Ko12
amhX+36
amhΔY-233
amhY-5608
Ld
Ld
1000bp
Ld
1000bp
800bp
Ld
Ld
8022bp
2414bp

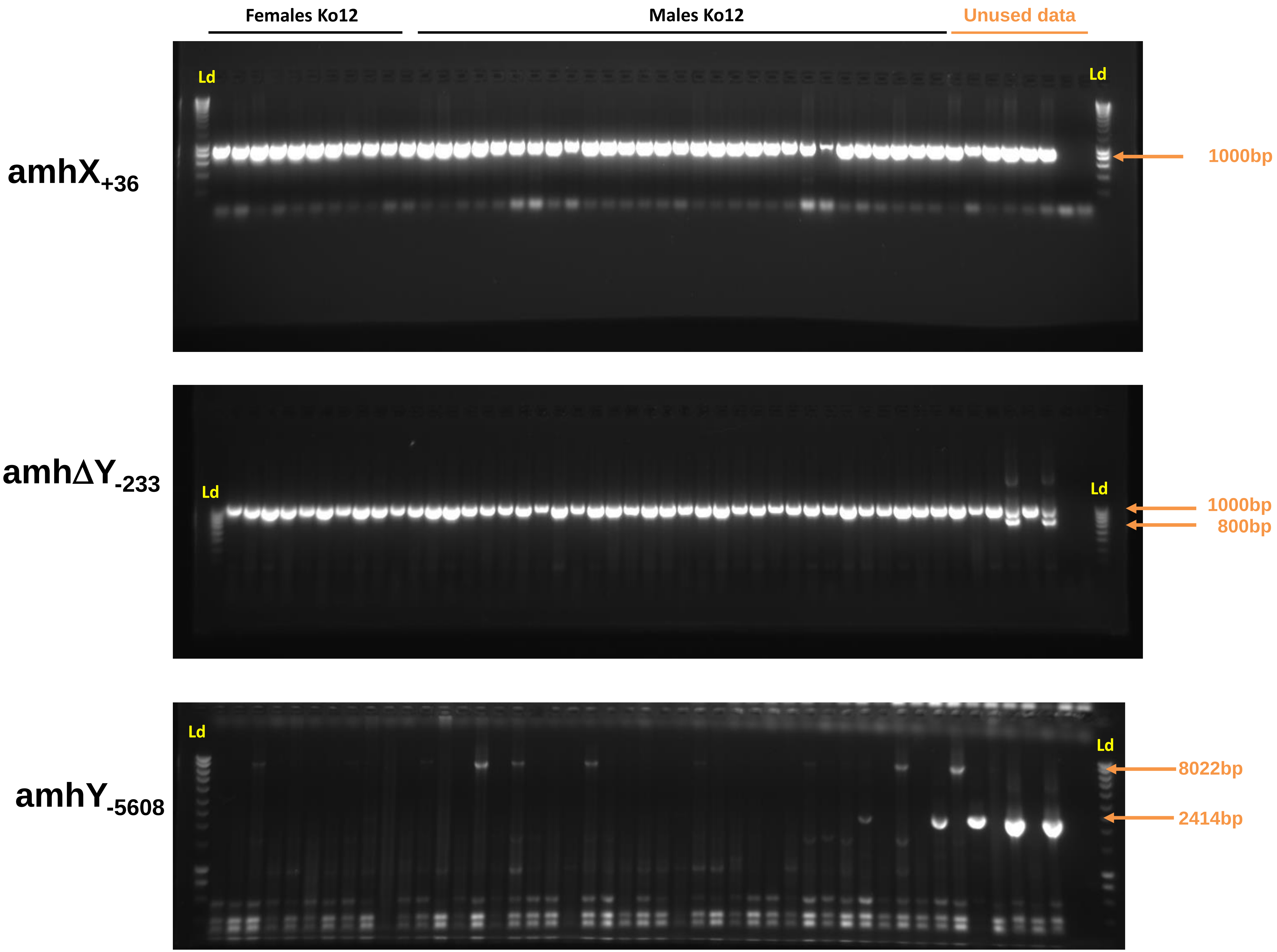
Females Ko12
Males Ko12
Unused data
amhX+36
Ld
Ld
1000bp
amhΔY-233
Ld
Ld
1000bp
800bp
amhY-5608
Ld
Ld
8022bp
2414bp

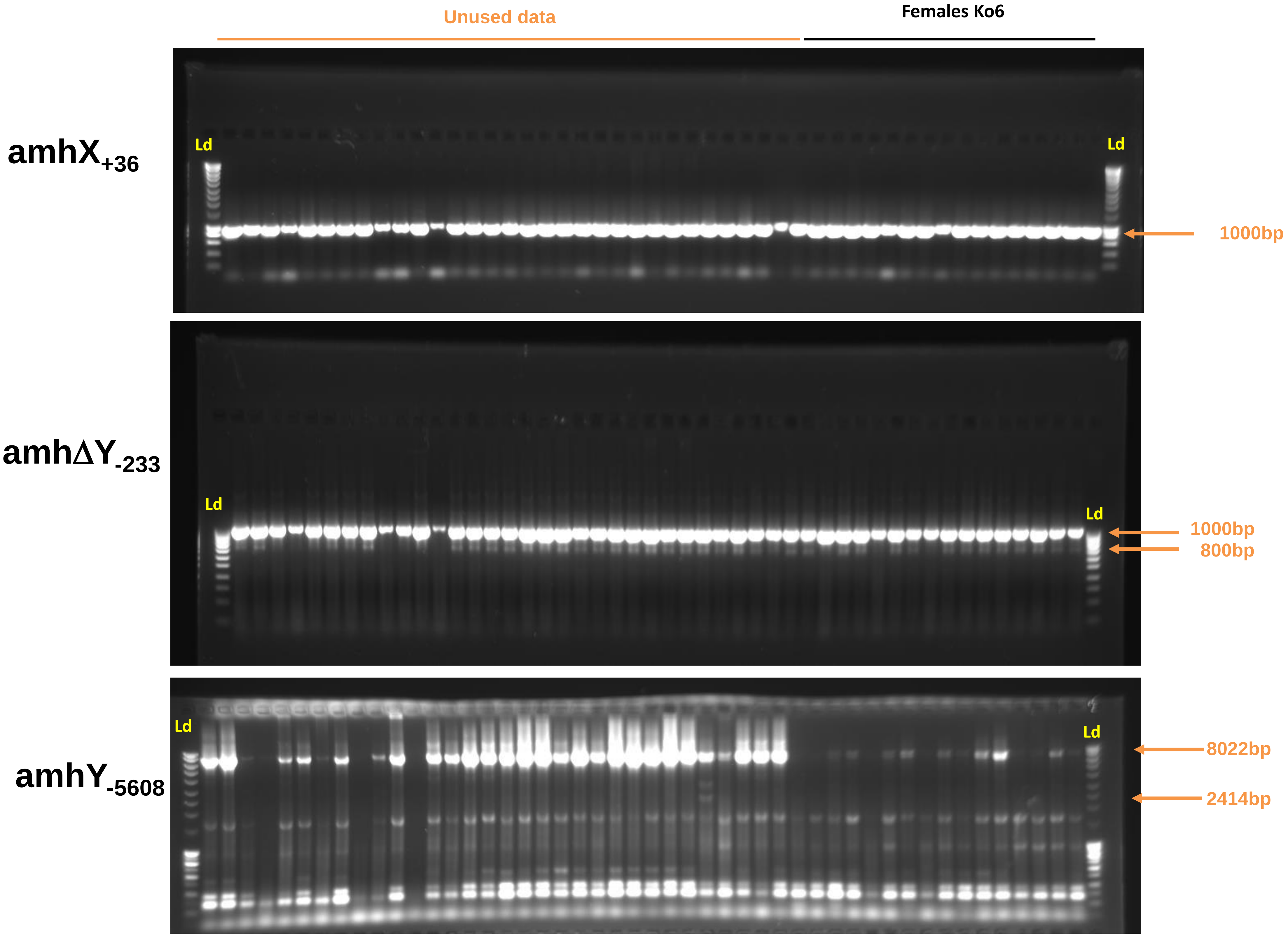
Unused data
Females Ko6
amhX+36
Ld
Ld
1000bp
amhΔY-233
Ld
Ld
1000bp
800bp
amhY-5608
Ld
Ld
8022bp
2414bp

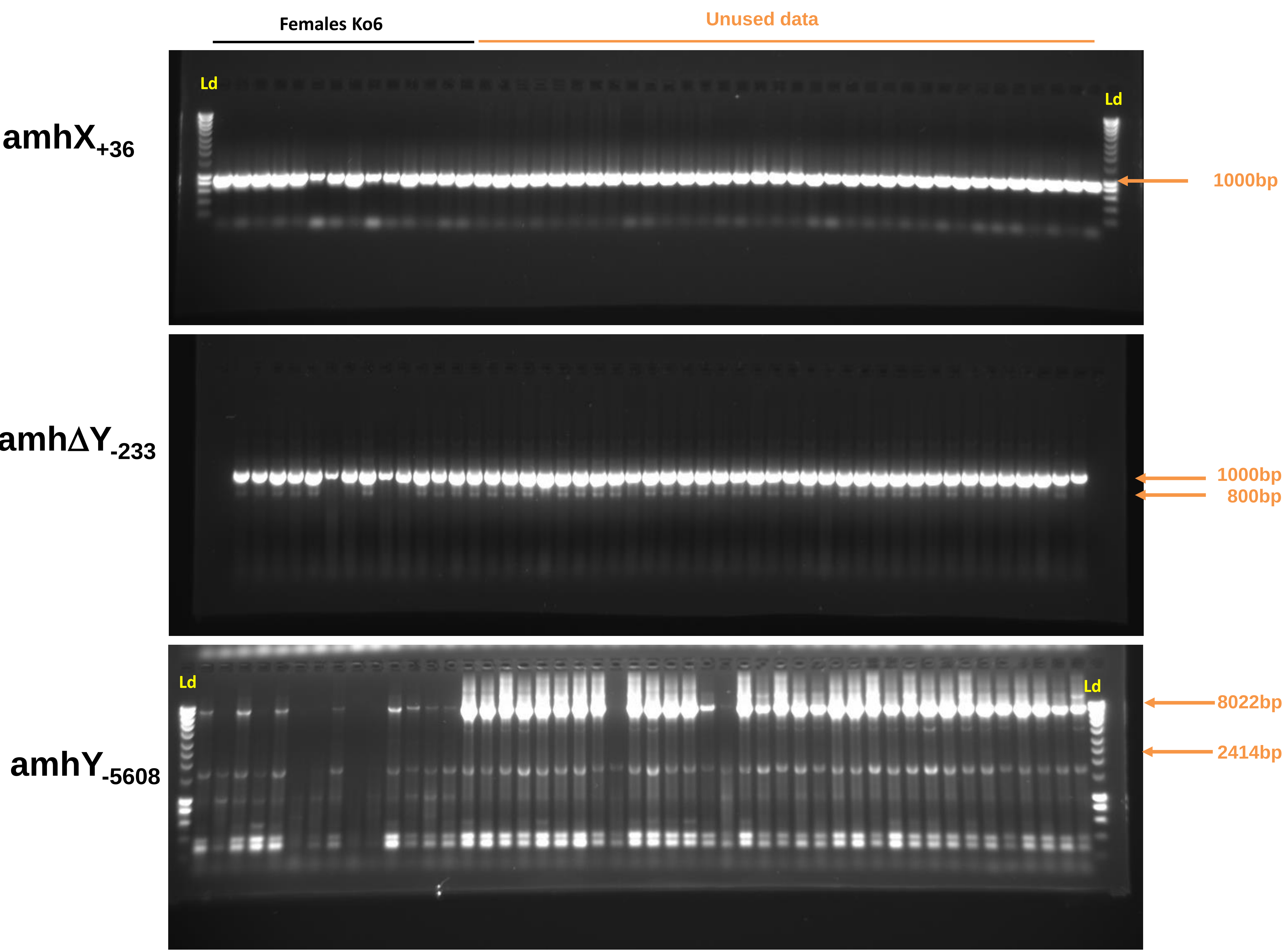
Females Ko6
Unused data
Ld
Ld
amhX+36
1000bp
amhΔY-233
1000bp
800bp
Ld
Ld
amhY-5608
8022bp
2414bp

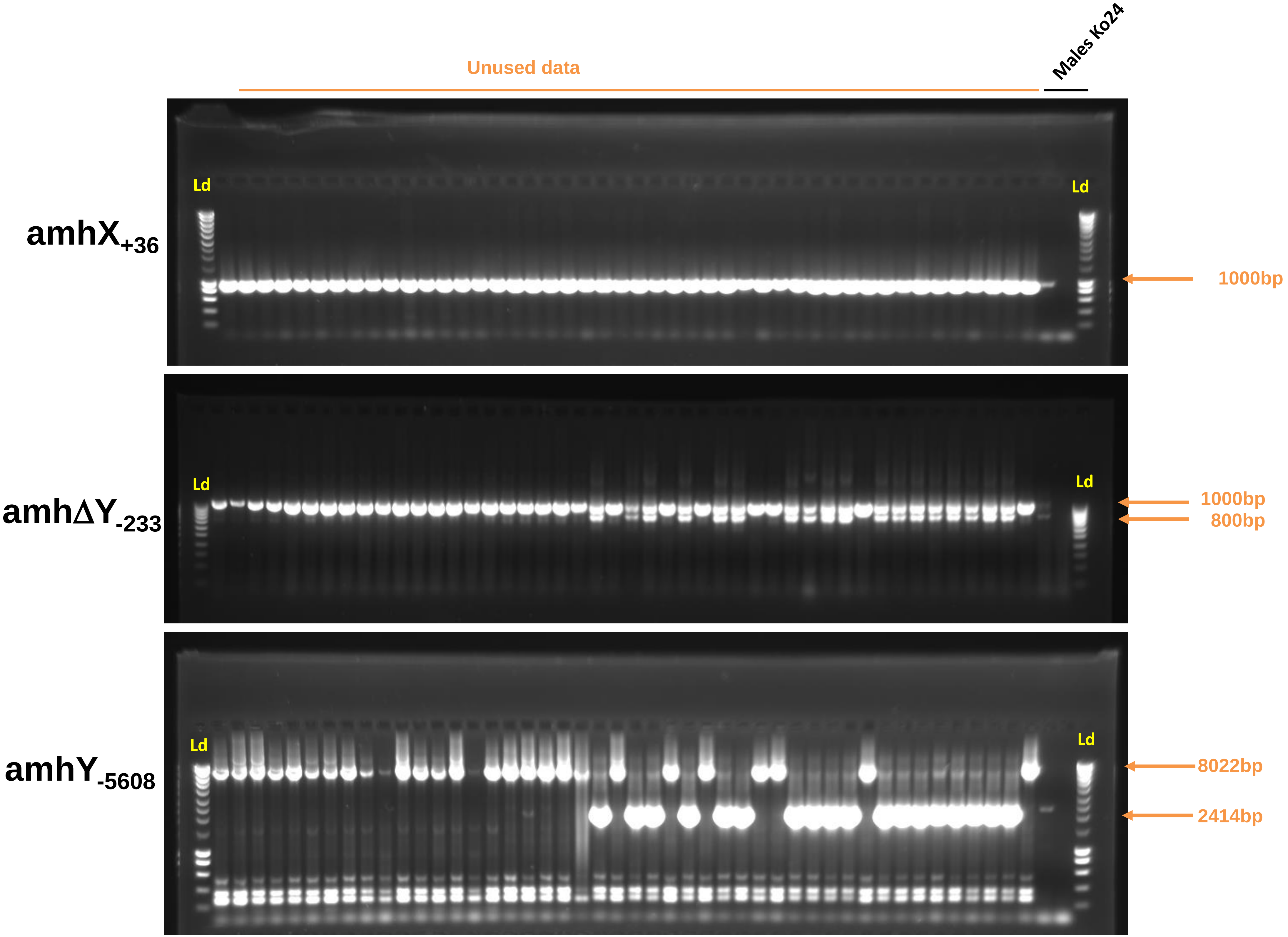
Unused data
Males Ko24
amhX+36
Ld
Ld
1000bp
amhΔY-233
Ld
Ld
1000bp
800bp
amhY-5608
Ld
Ld
8022bp
2414bp

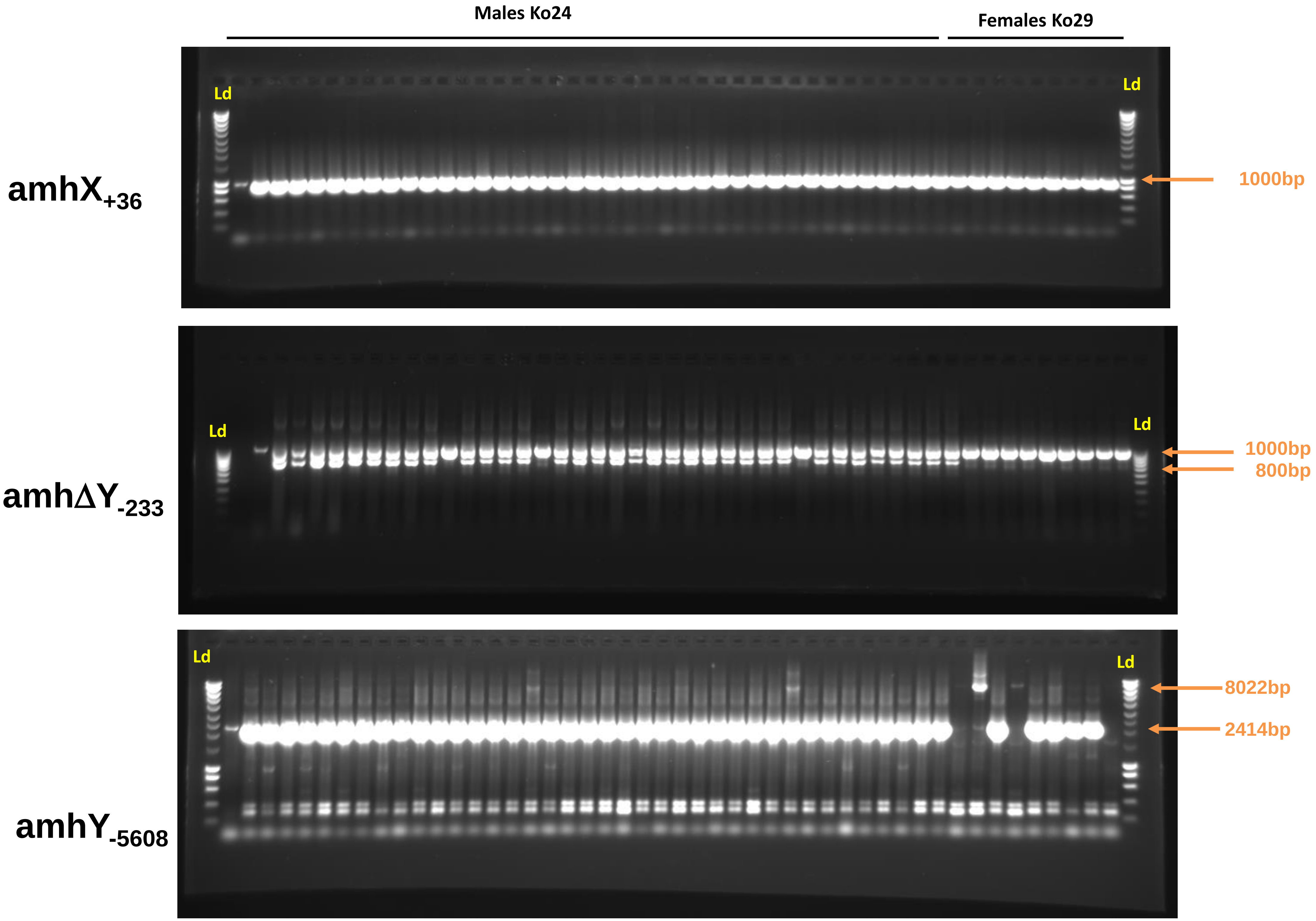

Males Ko24
Females Ko29
Ld
Ld
amhX+36
1000bp
Ld
Ld
amhΔY-233
1000bp
800bp
Ld
Ld
amhY-5608
8022bp
2414bp

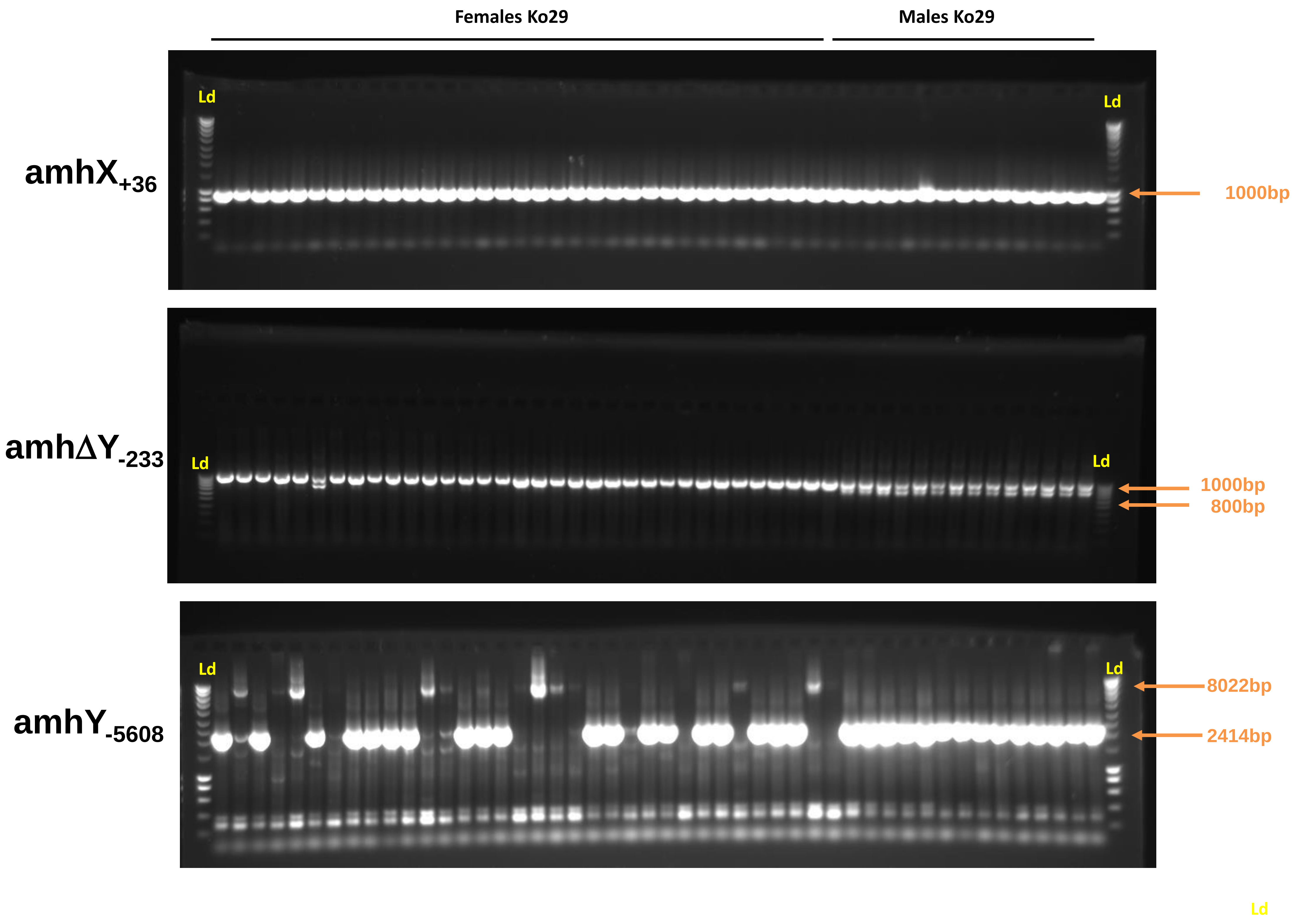
Females Ko29
Males Ko29
amhX+36
Ld
Ld
1000bp
amhΔY-233
Ld
Ld
1000bp
800bp
amhY-5608
Ld
Ld
8022bp
2414bp
Ld

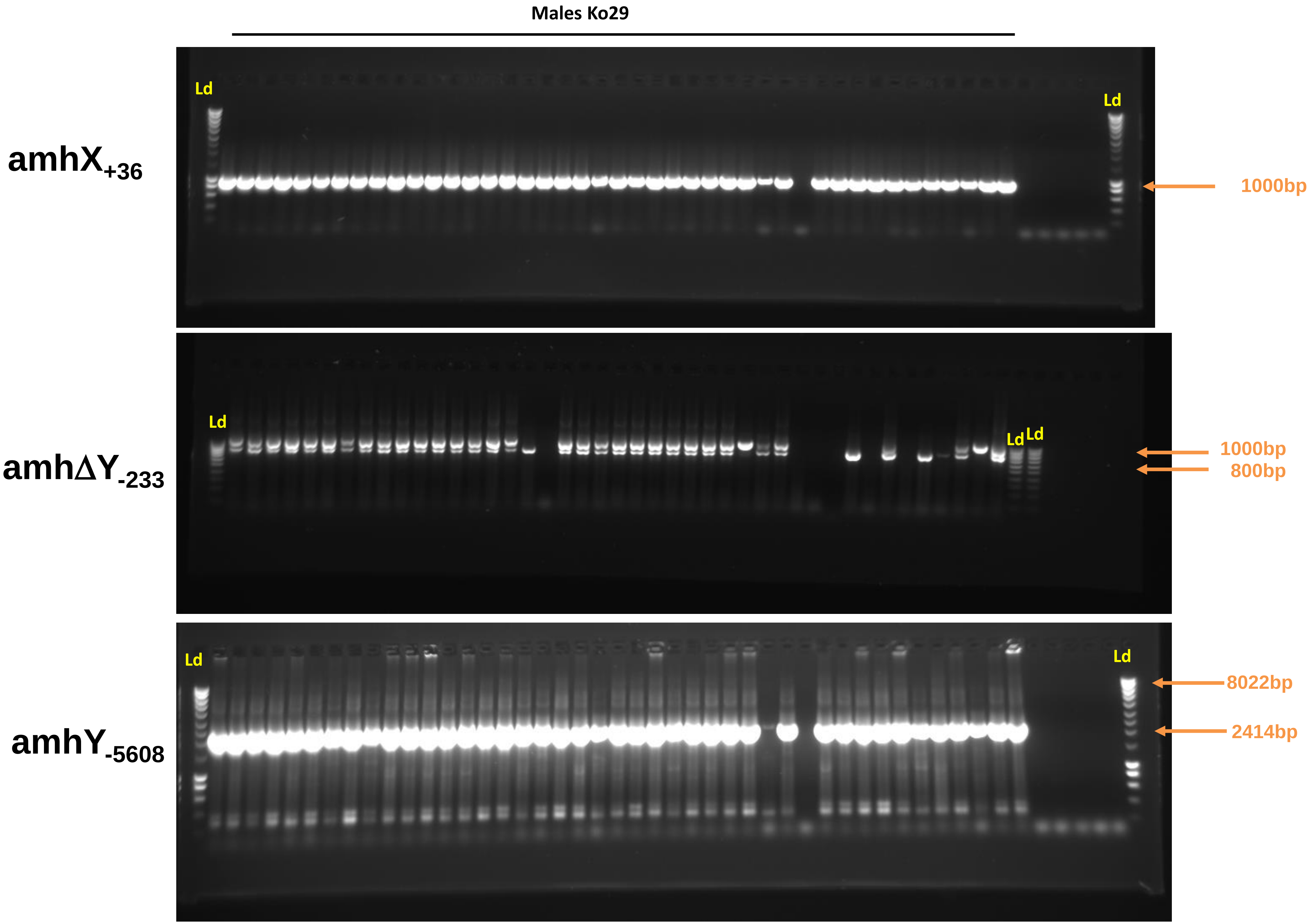
Males Ko29
Ld
Ld
amhX+36
1000bp
Ld
Ld Ld
amhΔY-233
1000bp
800bp
Ld
Ld
amhY-5608
8022bp
2414bp
